# St. Louis Encephalitis Virus in the Southwestern United States: A Phylogeographic Case for a Multi-Variant Introduction Event

**DOI:** 10.3389/fgene.2021.667895

**Published:** 2021-06-08

**Authors:** Chase L. Ridenour, Jill Cocking, Samuel Poidmore, Daryn Erickson, Breezy Brock, Michael Valentine, Chandler C. Roe, Steven J. Young, Jennifer A. Henke, Kim Y. Hung, Jeremy Wittie, Elene Stefanakos, Chris Sumner, Martha Ruedas, Vivek Raman, Nicole Seaton, William Bendik, Heidie M. Hornstra O’Neill, Krystal Sheridan, Heather Centner, Darrin Lemmer, Viacheslav Fofanov, Kirk Smith, James Will, John Townsend, Jeffrey T. Foster, Paul S. Keim, David M. Engelthaler, Crystal M. Hepp

**Affiliations:** ^1^School of Informatics, Computing, and Cyber Systems, Northern Arizona University, Flagstaff, AZ, United States; ^2^The Pathogen and Microbiome Institute, Northern Arizona University, Flagstaff, AZ, United States; ^3^Translational Genomics Research Institute, Flagstaff, AZ, United States; ^4^Vector Control Division, Maricopa County Environmental Services Department, Phoenix, AZ, United States; ^5^Coachella Valley Mosquito and Vector Control District, Indio, CA, United States; ^6^Yuma County Pest Abatement District, Yuma, AZ, United States; ^7^Southern Nevada Health District, Las Vegas, NV, United States

**Keywords:** St. Louis encephalitis virus, BEAST, genomics, phylogenetic analysis, Culex mosquitoes

## Abstract

Since the reemergence of St. Louis Encephalitis (SLE) Virus (SLEV) in the Southwest United States, identified during the 2015 outbreak in Arizona, SLEV has been seasonally detected within *Culex* spp. populations throughout the Southwest United States. Previous work revealed the 2015 outbreak was caused by an importation of SLEV genotype III, which had only been detected previously in Argentina. However, little is known about when the importation occurred or the transmission and genetic dynamics since its arrival into the Southwest. In this study, we sought to determine whether the annual detection of SLEV in the Southwest is due to enzootic cycling or new importations. To address this question, we analyzed 174 SLEV genomes (142 sequenced as part of this study) using Bayesian phylogenetic analyses to estimate the date of arrival into the American Southwest and characterize the underlying population structure of SLEV. Phylogenetic clustering showed that SLEV variants circulating in Maricopa and Riverside counties form two distinct populations with little evidence of inter-county transmission since the onset of the outbreak. Alternatively, it appears that in 2019, Yuma and Clark counties experienced annual importations of SLEV that originated in Riverside and Maricopa counties. Finally, the earliest representatives of SLEV genotype III in the Southwest form a polytomy that includes both California and Arizona samples. We propose that the initial outbreak most likely resulted from the importation of a population of SLEV genotype III variants, perhaps in multiple birds, possibly multiple species, migrating north in 2013, rather than a single variant introduced by one bird.

## Introduction

St. Louis Encephalitis Virus (SLEV) is the causative agent of the disease St. Louis Encephalitis (SLE). Transmission of SLEV is enzootically cycled between *Culex* spp. mosquito vectors and numerous bird hosts (Day, 2001; Reisen, 2003). Although SLEV can infect humans, it does not achieve high enough levels of viremia to be further transmitted; humans are a dead end host. SLE symptoms include headache, fever, nuchal rigidity, disorientation, and tremor, and are often confused with the flu. However, 80% of clinical cases result in encephalitis, of which 5–20% is fatal (Reisen, 2003). SLEV was not discovered until 1933, after a viral outbreak in St. Louis, Missouri resulted in 1,095 reported cases, including 201 fatalities (Webster and Fite, 1933). Since 1933, SLEV has been characterized into eight genotypes (I–VIII; Day, 2001; Kramer and Chandler, 2001; Baillie et al., 2008). The only genotypes known to cause disease in humans are genotypes I, II, and III, with genotypes I and II being endemic to North America, and genotype III formerly endemic only to South America (Day, 2001). Genotypes I and II have caused over 50 SLEV outbreaks in the United States and southern Canada, resulting in ~10,000 reported encephalitis cases and more than 1,000 fatalities (Webster and Fite, 1933; Day, 2001).

St. Louis Encephalitis Virus’s incidence and medical prominence within the United States were displaced with the importation of West Nile Virus (WNV) in 1999 (Lanciotti et al., 1999; Reisen et al., 2008). WNV quickly spread throughout the entire United States, becoming established by 2004 (Reisen, 2013). Concurrent with the geographic radiation of WNV, SLEV cases caused by genotypes I and II rapidly decreased (Reisen et al., 2008; Diaz et al., 2018). Both SLEV and WNV are flaviviruses, imparting cross-immunity in shared hosts (Tesh et al., 2002; Drummond and Rambaut, 2007). Previous efforts have revealed that prior infection with WNV can cause complete immunity to a secondary SLEV genotype II or V infection, whereas a primary SLEV infection reduced the viral load of a subsequent WNV infection by a 1,000-fold (Tesh et al., 2002; Brault et al., 2004; Fang and Reisen, 2006; Maharaj et al., 2018). It is quite plausible that these cross-immunity effects are responsible for the drastic decrease of SLEV cases caused by genotypes I and II after the rapid dispersal of WNV (Reisen et al., 2008).

In 2015, an SLEV outbreak occurred in Maricopa County, Arizona where 23 individuals were infected, 19 of who developed encephalitis, resulting in two fatalities (White et al., 2016; Maharaj et al., 2018). These were the first cases of SLEV in Arizona in 10 years. Moreover, this was the first outbreak in the United States since the 2001 Louisiana outbreak that resulted in four deaths (Jones et al., 2002). The strain isolated from the 2015 epidemic was related most closely to one previously circulating in Argentina, placing it within genotype III, which caused the first epidemic in South America (Diaz et al., 2006; White et al., 2016). During 2005, Cordoba City, Argentina had 47 probable human cases of SLEV, which resulted in 45 hospitalizations and nine deaths (Diaz et al., 2006). This is not the first time a genotype first detected in South America was observed circulating within the United States. An SLEV strain collected in Florida in 2006, determined to be genotype V, was previously observed only in South America (Kramer and Chandler, 2001; Ottendorfer et al., 2009). The current working hypothesis is that genotypes from South America have been introduced into North America *via* migratory birds.

To date, it is unknown whether seasonal SLEV in the Southwest are due to the virus overwintering locally or from annual importations from surrounding regions (Reisen et al., 2006; Diaz et al., 2018). Therefore, acquiring a thorough understanding of the molecular history and epidemiology of SLEV is essential for health agencies to assess the potential risk of SLEV to the local populations (Maharaj et al., 2018). The overarching goals of our study were to (1) determine when SLEV entered the Southwest United States, hereafter, referred to as the Southwest; (2) characterize the spatial-temporal trajectories of SLEV within Maricopa County, AZ and Riverside County, CA – two counties with a substantial annual SLEV burden; and (3) identify how SLEV variants within the two aforementioned counties are related to those in nearby counties.

## Materials and Methods

### Sample Collection

#### Maricopa County, Arizona

The Maricopa County Environmental Services Vector Control Division (MCESVCD) mosquito surveillance program places ~800 CO_2_ traps throughout the Phoenix Metropolitan area. Each trap is placed within its designated square mile area for a 12-h collection period once weekly for 50 weeks of the year (sampling is paused for epidemiological weeks 52 and 1).

#### Yuma County, Arizona

Yuma County Pest Abatement District (YCPA) has a surveillance season from March to late October or early November. During this time, YCPA places 22 Encephalitis Vector Survey (EVS) CO_2_ baited traps on a biweekly basis. The traps are placed in riparian areas in the early evening throughout the county and left for a 12 h collection period.

#### Coachella Valley, California

Coachella Valley Mosquito and Vector Control District (CVMVCD) conduct regular mosquito trapping and abatement. The CVMVCD splits surveillance in Coachella Valley into two regions: eastern valley and western valley. The eastern valley has 56 CO_2_ traps set every 2 weeks. The western valley has 53 gravid traps and 53 CO_2_ traps set weekly.

#### Clark County, Nevada

Southern Nevada Health District (SNHD) mosquito surveillance program has a surveillance season from March to through September. In 2019, they set, on average, 138 EVS CO_2_ baited traps per week (4,150 total) during the 7 month surveillance period. The traps are set throughout Clark County but the total number set per week is variable due to weather conditions or other Environmental Health responsibilities.

#### Mosquito Sorting and Identification

After collection, the mosquitoes were frozen in the field using dry ice. Mosquitoes were transported to the respective health agencies laboratory and sorted by sex, with males being discarded, while the females were pooled by species with a maximum of five pools per trap with 50 females per pool. Resulting pools were tested for WNV and SLEV, following the protocol described by Lanciotti et al. (1999) and Baillie et al. (2008). SLEV-positive pools were stored in −80°C freezers until they were either same-day transported to Northern Arizona University on dry ice or shipped using FedEx ground transportation while preserved in DNA/RNA Shield™ 2X Concentrate. Metadata supplied with each of the samples included the GPS coordinates of the mosquito traps, date of collection, total number of mosquitoes captured, and mosquito species. For this study, we selected 142 positive mosquito pools for whole genome sequencing, see Supplementary Table S1.

### Sample Processing, SLEV Tiled Amplicon Sequencing

The methods used to prepare SLEV samples for transport, RNA extraction, and reverse transcription followed the protocol previously described for WNV (Baillie et al., 2008; Hepp et al., 2018). Multiplex PCR primers were designed using the software package Primal Scheme (Quick et al., 2017); where the 42 primer pairs were based on an SLEV genome from Kern County, California (KY825743.1) with an average primer pair product of 400 bp, see Supplementary Table S2. For each sample, a multiplex PCR reaction was performed for each pool individually. The PCR reaction used 12.5 μl of KAPA 2G Fast Multiplex Mix (2X; Kapa Biosystems, Wilmington, MA, United States), with a final per primer concentration of 0.2 μM, 2.5 μl of cDNA, in a total reaction volume of 25 μl. The thermocycler settings used: 3 min of denaturation at 95°C, 30 cycles of 98°C for 15 s, 60°C for 30 s, and 72°C for 1 min, and a final extension of 72°C for 1 min. The PCR product was cleaned using 1X Agencourt AMPure XP beads (Beckman Coulter, Indianapolis, IN, United States). A second PCR using universal tail-specific primers was performed to add the Illumina specific indexes (Colman et al., 2015). The reagents for the reaction were 12.5 μl of 2X Kapa HiFi HotStart Ready Mix (Kapa Biosystems), 400 nM of each forward and reverse indexed primer, and 2 or 4 μl of the cleaned amplified SLEV product. The thermocycler protocol is as follows: 98°C for 2 min, 6 cycles of 98°C for 30 s, 60°C for 20 s, and 72°C for 30 s, and a final extension at 72°C for 5 min. The DNA for the samples in each pool was quantified using the Kapa Library Quantification kit (Kapa Biosystems). The samples were then pooled to achieve an equal concentration of each sample. Sequencing was conducted on the Illumina MiSeq sequencing platform, using a v3 600 cycle kit.

### Post-sequencing Data Processing

To generate consensus sequences needed for phylogenetic analysis, sequencing reads were first trimmed using Amplicon Sequencing Analysis Pipeline 0.9 (ASAP; https://github.com/TGenNorth/ASAP). ASAP trimmed reads of adapter and primer sequences using BBDuk, a tool integrated into the BBMap package.^1^ Resulting paired-trimmed reads were aligned to the reference genome, FJ753286.2, using Bowtie2 (Langmead and Salzberg, 2012), and alignments were indexed using Samtools 1.4.1 (Li et al., 2009). Consensus sequences were generated using the program iVar 1.0 (Li et al., 2009; Grubaugh et al., 2019). The criterion for base calling was a minimum of 10x coverage and a majority base proportion of 0.80. Base calls were coded according to the International Union of Pure and Applied Chemistry (IUPAC) nucleotide codes. Additionally, a read pileup was produced using the Integrated Genome Viewer (IGV) 2.4.16 command line tool (Robinson et al., 2017).

### Maximum Likelihood Analysis

To determine if SLEV strains circulating within Maricopa County and Coachella Valley were genotype III, we conducted a Maximum Likelihood phylogenetic analysis using the 74 publicly available whole genome sequences from the National Center for Biotechnology Information (NCBI), which includes representatives from genotypes I–VIII, and the 142 genomes sequenced by our lab. Sequences were aligned using MUSCLE (Edgar, 2004) followed by substitution model testing using IQ-TREE. The GTR substitution model with invariable sites and base frequencies sampled from a gamma distribution was determined to be the best fit model based on the Bayesian information criterion. The maximum-likelihood tree was constructed using IQ-TREE with 10,000 bootstraps iterations (Nguyen et al., 2015; Kalyaanamoorthy et al., 2017; Wang et al., 2018). The tree was rooted using the two strains of SLEV (JQ957869.1 and JQ957870.1) detected in Columbia in 2008 as described by Hoyos-López et al. (2015).

### Bayesian Phylogenetic Analysis

The temporal signal for the 174 SLEV genome dataset was determined by a root-to-tip genetic divergence and time of sampling regression, performed in TempEst v1.5.1 (Rambaut et al., 2016; Supplementary Figures S1A,B). TempEst requires a phylogeny where branch lengths are scaled by genetic distances as input. For our analysis, a maximum likelihood tree was generated by IQ-TREE using the TN93+𝚪 substitution model; the substitution model was the best fit to the data according to IQ-TREE’s model selection tool. It should be noted that the Arizona 2014 sample (KX965720) was removed from further analyses because it was an incomplete genome that had a drastically different temporal signal than the rest of the samples (i.e., an outlier, Supplementary Figure S1A, teal circle). Removing the 2014 Arizona sample improved the *R*^2^ value from 0.1 to 0.7, indicating that the maximum likelihood phylogeny branch lengths were positively correlated with time.

To estimate the time of entry and population structure of SLEV in the American Southwest, a Bayesian phylogenetic analysis was conducted using the BEAST v1.10.5 software package (Rambaut et al., 2016; Suchard et al., 2018). The specified substitution model, as determined by IQ-TREE previously, is the TN93+𝚪 nucleotide substitution model. The best fitting molecular clock and demographic models were determined by marginal likelihood comparison using path-sampling and stepping-stone sampling (Baele and Lemey, 2013; Baele et al., 2013; Supplementary Table S1). The Bayesian Skyline model was found to be best fitting in combination with a relaxed molecular clock. The final model was run on four independent Markov chains where each chain ran for 100 million Markov chain Monte Carlo steps, and the state was sampled every 10,000 steps. Convergence was assessed using Tracer v1.7 (Rambaut et al., 2016, 2018; Suchard et al., 2018). States were combined using LogCombiner, discarding the first 10% as burn-in (10,000,000 generations per chain), and then resampling every 30,000 generations. Trees were summarized using TreeAnnotator (Drummond and Rambaut, 2007), producing a maximum clade credibility tree that was visualized using the R package GGtree (Yu et al., 2017, 2018; Yu, 2020). The branches of the tree were collapsed into polytomies if the posterior support was below 0.50, using a custom R script https://github.com/ChaseR34.

### Phylogeny-Trait Association Analysis

To detect evidence of SLEV phylogenetic structure by Culex sp. vector, we inspected the Bayesian posterior sets of trees, produced by BEAST, using the Bayesian Tip-association Significance testing (BaTS) program (Parker et al., 2008). Briefly, BaTS uses three statistics, the Association Index (AI; Wang et al., 2001), Fitch parsimony score (PS; Fitch, 1971), and the maximum exclusive single-state clade size (MC; Parker et al., 2008), to determine if character states are clustered more frequently within a phylogeny than would be expected due to chance. We focused this analysis on the Clade 2 Maricopa County-derived genomes, 75 genomes in total, as that is the only location where SLEV positive mosquito pools were distributed among both *Culex quinquefasciatus* and *Cx. tarsalis* populations.

## Results

### Genetic Relatedness of 2015 to Historical SLEV Samples

The 142 genomes we sequenced were genotype III, as they all nested within the Argentinian samples (Supplementary Figure S1). Swetnam et al. (2020) sequenced 30 SLEV genomes from the Southwestern United States sampled from 2015 to 2018 and similarly found them to be genotype III. Therefore, our results corroborate Swetnam et al. (2020) along with previous work by White et al. (2016) and Diaz et al. (2018).

### BEAST Phylogenetic Analysis

#### Date of Entry and Bulk Migration Event

**Figure 1:**
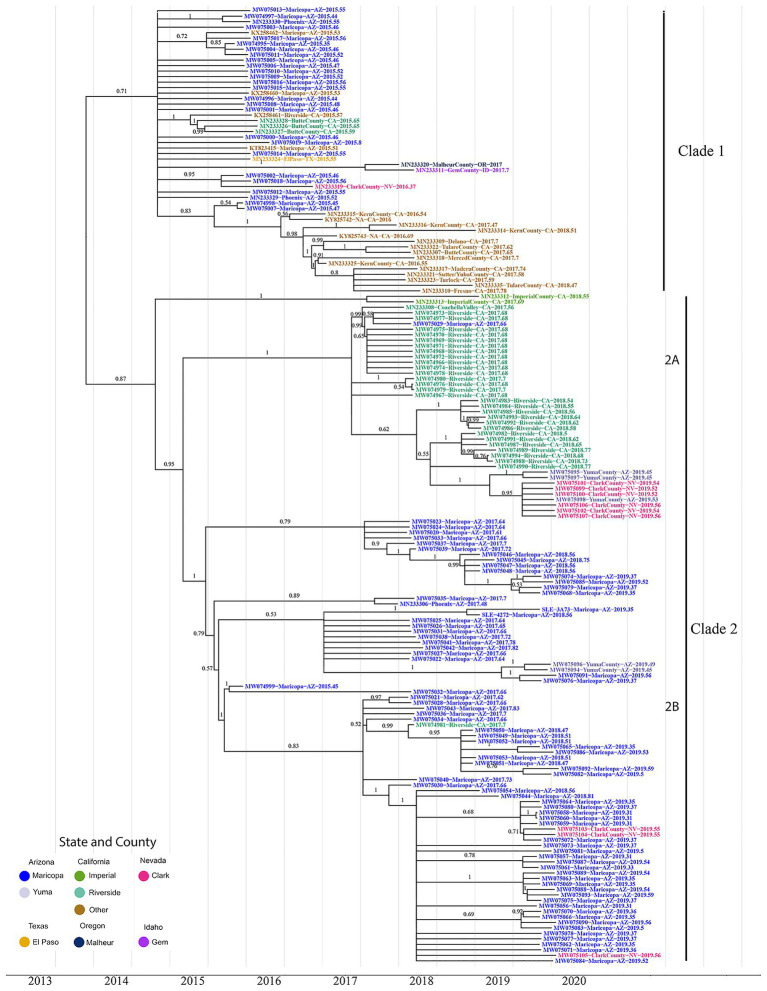
The maximum clade credibility phylogenetic tree reconstructed using 174 genotype III St. Louis Encephalitis Virus (SLEV) genomes throughout the Southwest. The tip colors distinguish the sampling location of each sample Arizona Counties, Maricopa (Blue), and Yuma (Gray); California Counties, Imperial (Green), Riverside (Teal), and Others (Brown); Nevada, Clark (Pink); Texas, El Paso (Yellow); Oregon, Malheur (Dark Blue); and Idaho, Gem (Purple). Posterior values for branches above 0.50 are written above their corresponding branch. Branches with posterior values below 0.5 were collapsed into polytomies (Supplementary Figure S3 contains the BEAST tree prior to collapsing and contains the 95% HPD CI for each node). After correcting for low confidence branches, the phylogeny clusters into three distinct clades. Clade 1 consists of all the 2015–2016 California and 2015 Arizona samples. Clade 2 consists of the 2017–2018 California samples and the 2017–2019 Arizona samples. Clade 2 is further divided into two subclades 2A and 2B. Subclade 2A comprises Coachella Valley, California; Yuma County, Arizona, and Clark County, Nevada; subclade 2B comprises 2017–2019 Arizona samples, with two Yuma County and three Clark County samples.

The first line of inquiry was to estimate the timing and location for the initial SLEV genotype III migration into the Southwest using BEAST. The phylogeny indicated two well-supported sister clades, here, well-supported means that the posterior probability of the clade being observed is over 0.5; hereafter, referred to as Clade 1 and Clade 2, see Figure 1. The formation of sister clades is indicative of multiple migration events into the Southwest, therefore the time of arrival needs to be addressed for each clade independently.

Clade 1, the most geographically diverse clade, contains samples from Arizona, California, Nevada, Oregon, Idaho, and Texas. Posterior estimates for branches below 0.5 were collapsed into polytomies (Figure 1; Supplementary Figure S2), which are indicative of a rapid radiation event (Whitfield and Lockhart, 2007). Moreover, Clade1 contains genomes from both Arizona and California where most California samples clustered monophyletically, with samples collected in 2017 and 2018 being sourced by variants circulating within the state in previous years. The remaining California, Arizona, and Nevada genomes do not cluster with genomes originating from the same location in downstream years. These data indicate that Clade I variants did not give rise to contemporary counterparts (i.e., 2017–2019) sequenced as part of this study. Finally, there is no well-supported basal and paraphyletic cluster of genomes from Arizona or California, and therefore, we are unable to determine if Clade I members of SLEV genotype III arrived into California or Arizona first. However, the lack of clear structure within Clade 1 leads us to consider the possibility that the introduction of SLEV into the Southwestern United States was due to migration over a short period of time. In this scenario, a flock or flocks of birds with multiple infected individuals may have entered the Southwest, explaining the lack of a robust signal supporting the arrival into one location over another.

Clade 2 is sister to Clade 1 with its topology containing a distinct basal node with the remaining samples nested within it. Two samples (MN233312 and MN233313) from Imperial Valley, California are at the base of the clade, with two distal subclades encompassing genomes from (2A) Coachella Valley, CA, to (2B) Maricopa County, CA. Coachella Valley is immediately north of Imperial Valley, and the two counties share the Salton Sea where many of the Coachella Valley samples sequenced in this study were collected. Taken together, southern California genomes form a basal and paraphyletic cluster in relationship to Maricopa County genomes (Figure 1), Clade (2A) providing evidence for a second migration event originating within California and spreading to Maricopa County and other parts of the Southwest.

#### Characterizing Source Location of Seasonal SLEV Populations

Our second line of inquiry was to determine whether seasonal occurrences of SLEV within Maricopa County, Arizona, and Coachella Valley, California are sourced from endemically circulating variants or are newly introduced each year *via* annual migration events. Because we are interested in estimating migration patterns for current populations, only the two clades containing extant lineages, Clade 2A composed primarily of Coachella Valley samples and Clade 2B containing Maricopa County samples, were considered. The geographic clustering allows us to draw two conclusions: first, migration events between Coachella Valley and Maricopa County have been rare, and second, the inference about the two populations can be done independently. Therefore, the two clades are interpreted separately below.

Clade 2A is estimated to have originated in late 2016 (2016.9, 95% HPD CI:2015.9–2017.2), and contains Coachella Valley, California samples from 2017 to 2018. The internal branch topology of Clade 2 supports two monophyletic clusters, distinguished by year of collection. This topology is indicative of two possible scenarios for the seasonal appearance of SLEV. The first scenario is that the population of SLEV in 2018 was seeded by cryptically circulating SLEV from the previous year. The second and more plausible scenario is that a closely related, and perhaps closely situated and endemic, population of SLEV was imported into Coachella Valley each year.

Clade 2B, estimated to have originated in 2014 to early 2015 (2015.0, 95% HPD CI:2014.0–2015.1), is composed of genomes sequenced from samples collected in Arizona in 2015 (a single sample), 2017, 2018, and 2019, and a single California genome. There are two plausible patterns to the seasonal establishment of SLEV within Maricopa County. First, if SLEV was sourced from nearby regions rather than internally, we would expect to observe multiple monophyletic clades that cluster by year, mirroring the Clade 2A topology. The second possibility is a temporally paraphyletic clade that includes the 2018 and 2019 Arizona samples nesting within the 2017 Arizona samples. The phylogeny clearly shows that Clade 2A is consistent with the second scenario. Therefore, our results indicate SLEV has become endemic in Maricopa County and the seasonal emergence of SLEV is due to annual re-seeding by multiple local populations.

#### Movement Within the Southwest United States

Clade 1 contains all but one of the genomes from samples collected in 2015, and the majority forms a polytomy, indicative of rapid population expansion or the presence of multiple seeding events with SLEV genotype III over a short period of time in the Southwest. Given this polytomy, there is not enough posterior support to determine in which location this genotype first entered. In addition, none of the Clade 1 genomes sampled in 2015 have clear extant descendants. However, there is a cluster of genomes from samples collected in 2016–2018 in Central California, although their origination location is unclear. Genomes from two samples collected in Idaho and Oregon in 2017, which cluster together, are also not nested within sequences from any other state, indicating that samples currently available from 2015 do not capture the diverse population of SLEV that was present early on. Also, the Texas sample is part of the polytomy, which may indicate that this diverse migration event expanded farther east than had been previously considered. It is estimated that Clade 1 members share a common ancestor that migrated into the Southwest around July 2013 (2013.5, 95% HPD CI:2011.7–2014.7).

Within clade 2, Maricopa County and Coachella Valley form sister clades. This provides evidence that migrations between California and Maricopa County are very rare, as there is only a single Maricopa County genome within Clade 2A and a single Coachella Valley genome within Clade 2B. The Coachella Valley clade forms two sister clades that are segregated temporally, providing evidence that SLE is not endemic to Coachella Valley but outbreaks are caused by seasonal migration events into it from a nearby source location such as Imperial County. The 2018 sister clade has Clark County and Yuma County samples nested within it, indicating migration from Coachella Valley to these two counties.

The Maricopa County sister clade is composed of a single 2015 and several 2017, 2018, and 2019 samples. Each year is nested within the previous year, indicating that SLEV has become endemic in Maricopa County, as it has in central and northern California (Swetnam et al., 2020). The formation of polytomies within each year is most likely the result of oversampling as described for clade 1. Furthermore, Yuma and Clark County samples nesting within Maricopa County samples highlight likely migration events out of Maricopa County.

### Phylogenetic Structure by Vector

To determine if SLEV variants are structured within the Bayesian phylogeny by Culex vector, we performed an analysis using the program BaTS (Parker et al., 2008). The BaTS analysis with 1,000 null model replicates resulted in all four test statistics failed to reject the null hypothesis that SLEV’s mosquito vectors, *Cx. quinquefasciatus* and *Cx. tarsalis*, are randomly distributed throughout the phylogeny. Therefore, we conclude that we are unable to detect any phylogenetic-trait associations between SLEV and its mosquito vectors (Table 1).

**Table 1:** The results from the four test statistics used by bayesian tip-association significance testing (BaTS) to determine if there were phylogenetic-trait associations between SLEV and its mosquito vectors *Culex quinquefasciatus* and *Cx. tarsalis*.

Statistic	Observed mean	Lower 95% CI	Upper 95% CI	Null mean	Lower 95% CI	Upper 95% CI	Significance
AI	3.923	3.061	4.790	3.584	2.792	4.347	0.754
PS	26.704	24.000	29.000	24.727	21.604	27.443	0.917
MC (state 0)	2.490	2.000	3.000	2.920	2.188	4.226	0.997
MC (state 1)	3.177	2.000	5.000	3.855	2.760	6.257	0.859

## Discussion

The focus of our study was to better understand the dynamics of SLEV circulation in the southwestern United States since the 2015 outbreak. We sequenced 142 genomes from Coachella Valley, CA, Maricopa County, AZ, Yuma County, AZ, and Clark County, NV from 2015 through 2019. Using Bayesian phylogenetic techniques, we considered the following: (1) the timing of the first introduction of genotype III into the Southwest, (2) the number of distinct introductions, (3) whether contemporary variants in Maricopa County or Coachella Valley have become endemic, and (4) the amount of time endemic variants have been established.

Our study revealed that the introduction of SLEV into the Southwest occurred between September 2011 and May 2014, but went undetected until the 2015 outbreak. Unfortunately, it is currently unknown how the importation of SLEV into the Southwest occurred. To address this question would require substantially more SLEV positive mosquito samples and bird competence studies from Central and South America. Therefore, we were unable to confidently provide any scenarios of importation but we were able to address the question of SLEV dynamics pre and post 2015 outbreak within Maricopa County and Coachella Valley.

Since the 2015 outbreak, SLEV has been reliably circulating within the region. However, an interesting caveat occurred in 2016 where Maricopa County, the epicenter of the 2015 outbreak, reported zero human cases and no positive mosquito traps. It is unclear why SLEV was not observed in 2016, even though testing in Maricopa County, AZ did not change between 2015 and 2016. However, in 2017, SLEV was detected at high levels again and has been consistently detected each year since.

**Figure 2:**
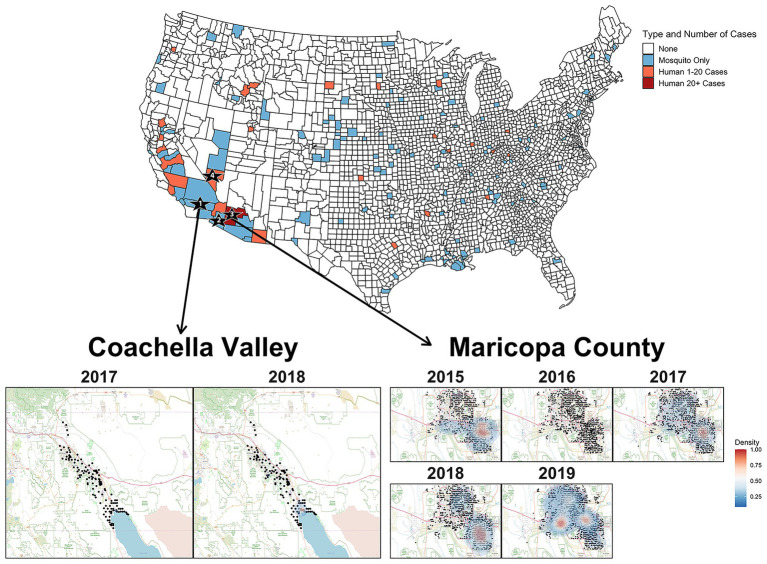
The United States map colors counties where SLEV positive mosquitoes or human cases were observed between 2015 and 2019. Sampling locations for the genomes sequenced in this study are indicated by the numbered black stars. Star 1 is Coachella Valley, Star 2 is Yuma County, Star 3 is Maricopa County, and Star 4 is Clark County. Heat map of densities of positive mosquito traps found within Coachella Valley from 2015 to 2018 and Maricopa County from 2015 to 2019. The black points represent the individual mosquito trap locations. Two-dimensional kernel density estimation with axis-aligned bivariate normal kernel was used to estimate the density of positive mosquito traps within each year. Red indicates a higher density of positive traps.

The phylogeny in Figure 2 strongly suggests that SLEV had at least two introductions into the Southwest. Therefore, the reemergence of SLEV in Maricopa County, Arizona was reestablished by a second introduction, independent of the primary 2015 outbreak introduction, which established populations in California and Arizona. Clades 2A (California) and 2B (Arizona) are isolated populations with limited migration between the two, so it seems unlikely that a migration event from Coachella seeded the 2017 reemergence in Maricopa County. This is not the first time that a SLEV genotype has had multiple introductions into the southwestern United States. In 2001 and 2002, genotype V was detected in Southern California and the samples formed temporally separated sister clades, similar to the pattern observed in Coachella for 2017 and 2018 (Reisen et al., 2002; Auguste et al., 2009). Finally, we found that SLEV does not have a vector bias which implies that both *Cx. quinquefasciatus* and *Cx*. *tarsalis* play pivotal roles in the seasonal circulation of SLEV within Maricopa County, Arizona.

Unlike Maricopa County, it is unclear whether the seasonal circulation of SLEV in Coachella Valley is seeded by the immigration of SLEV from a nearby endemic population or if there is endemic low-level circulation within Coachella Valley. Efforts prior to the emergence of SLEV genotype III revealed that previous genotypes overwintered in a saltmarsh habitat adjacent to the northeastern edge of the Salton Sea, then spread along the Salton Sea, and northwest into Coachella Valley (Reisen et al., 1995a,b; Reisen and Wheeler, 2016). From 1996 to 2013, Reisen and Wheeler (2016) captured 28,388 birds and collected serum samples, finding that individuals from 15 species were seropositive for SLEV in Coachella Valley. It is possible that these species may also participate in reseeding Coachella Valley from an endemic SLEV genotype III population maintained at the Salton Sea. However, a more complete sampling of potential avian hosts is necessary to determine which species are participating in short-distance migration events responsible for SLEV genotype III movement between sites in this region. We suggest focusing efforts on species that were previously identified as seropositive (Reisen and Wheeler, 2016), or those that have been identified through bloodmeal analyses, as previous studies have had relatively limited sample sizes for many species despite many decades of sampling for viral encephalitides (Lord et al., 1974; Reisen et al., 2003, 2005; Diaz et al., 2016; Pedersen et al., 2016; Díaz et al., 2018). Furthermore, the formation of monophyletic clades for 2017 and 2018 within Clade 2A, leads us to tentatively conclude that transient variants of SLEV are responsible for the seasonal circulation within Coachella Valley (Reisen et al., 2002). However, samples from other regions of California are needed to conclusively determine the origin of Coachella Valley’s seasonal SLEV populations.

Overall, SLEV genotype III has been the most frequently identified genotype in human cases since its detection in 2015. Between 2015 and September 2019, 50 of 59 human cases of SLEV reported nationwide to the Center for Disease Control (CDC) were genotype III and occurred in Arizona and California. Furthermore, in 2019, the CDC reported the highest SLEV mosquito infections ever recorded in the southwestern United States. In Maricopa County, this phenomenon occurred alongside WNV, which also had a near record year for detections in 2019. Coachella Valley had unprecedented detection levels of both viruses as well, with 513 WNV and 105 SLEV positive samples. Such co-circulation magnitude for SLEV and WNV has not been previously observed in the United States. Therefore, like WNV, SLEV appears to be an emerging health risk in the southwestern United States.

## Data Availability Statement

The datasets presented in this study can be found in online repositories. The names of the repository/repositories and accession number(s) can be found in the article/Supplementary Material.

## Author Contributions

CRi and CH contributed to conception and design of the study and wrote a first draft of the manuscript. CH acquired the funding for the project. SP, DEr, BB, and MV assisted in processing the samples and updated the database. HH, KSh, and HC sequenced the samples and developed the relevant protocols. SY, KSm, JWt, JWl, and JT provided the lab resources, samples, extracted RNA, and crucial revisions on the manuscript. JH, KH, JWt, JWl, ES, CS, MR, VR, NS, and WB provided the lab resources, collected mosquitoes, extracted samples, and crucial revisions on the manuscript. DL and VF assisted with post-processing of the sequence data. JF, CRo, PK, and DEn provided the expertise knowledge that assisted in interpreting phylogenetic results and their broader impacts. PK and DEn provided the laboratory space and equipment. All authors contributed extensively to the editing process. All authors contributed to the article and approved the submitted version.

### Conflict of Interest

The authors declare that the research was conducted in the absence of any commercial or financial relationships that could be construed as a potential conflict of interest.

## Footnotes

^1^https://sourceforge.net/projects/bbmap/

## References

[ref1] Auguste A. J. Pybus O. G. Carrington C. V. F. (2009). Evolution and dispersal of St. Louis encephalitis virus in the Americas. Infect. Genet. Evol. 9, 709–715. 10.1016/j.meegid.2008.07.006, PMID: 18708161

[ref2] Baele G. Lemey P. (2013). Bayesian evolutionary model testing in the phylogenomics era: matching model complexity with computational efficiency. Bioinformatics 29, 1970–1979. 10.1093/bioinformatics/btt340, PMID: 23766415

[ref3] Baele G. Lemey P. Vansteelandt S. (2013). Make the most of your samples: bayes factor estimators for high-dimensional models of sequence evolution. BMC Bioinformatics 14:85. 10.1186/1471-2105-14-85, PMID: 23497171 PMC3651733

[ref4] Baillie G. J. Kolokotronis S.-O. Waltari E. Maffei J. G. Kramer L. D. Perkins S. L. (2008). Phylogenetic and evolutionary analyses of St. Louis encephalitis virus genomes. Mol. Phylogenet. Evol. 47, 717–728. 10.1016/j.ympev.2008.02.015, PMID: 18374605

[ref5] Brault A. C. Langevin S. A. Bowen R. A. Panella N. A. Biggerstaff B. J. Miller B. R.. (2004). Differential virulence of West Nile strains for American crows. Emerg. Infect. Dis. 10, 2161–2168. 10.3201/eid1012.040486, PMID: 15663854 PMC1237116

[ref6] Colman R. E. Schupp J. M. Hicks N. D. Smith D. E. Buchhagen J. L. Valafar F.. (2015). Detection of low-level mixed-population drug resistance in *Mycobacterium tuberculosis* using high fidelity amplicon sequencing. PLoS One 10:e0126626. 10.1371/journal.pone.0126626, PMID: 25970423 PMC4430321

[ref7] Day J. F. (2001). Predicting St. Louis encephalitis virus epidemics: lessons from recent, and not so recent, outbreaks. Annu. Rev. Entomol. 46, 111–138. 10.1146/annurev.ento.46.1.111, PMID: 11112165

[ref8] Diaz A. Coffey L. L. Burkett-Cadena N. Day J. F. (2018). Reemergence of St. Louis encephalitis virus in the Americas. Emerg. Infect. Dis. 24, 2150–2157. 10.3201/eid2412.180372, PMID: 30457961 PMC6256408

[ref9] Díaz A. Flores F. S. Quaglia A. I. Contigiani M. S. (2018). Evaluation of Argentinean bird species as amplifying hosts for St. Louis encephalitis virus (Flavivirus, Flaviviridae). Am. J. Trop. Med. Hyg. 99, 216–221. 10.4269/ajtmh.17-0856, PMID: 29761767 PMC6085794

[ref10] Diaz L. A. Quaglia A. I. Konigheim B. S. Boris A. S. Aguilar J. J. Komar N.. (2016). Activity patterns of St. Louis encephalitis and West Nile viruses in free ranging birds during a human encephalitis outbreak in Argentina. PLoS One 11:e0161871. 10.1371/journal.pone.0161871, PMID: 27564679 PMC5001705

[ref11] Diaz L. A. Ré V. Almirón W. R. Farías A. Vázquez A. Sanchez-Seco M. P.. (2006). Genotype III Saint Louis encephalitis virus outbreak, Argentina, 2005. Emerg. Infect. Dis. 12, 1752–1754. 10.3201/eid1211.060486, PMID: 17283629 PMC3372344

[ref12] Drummond A. J. Rambaut A. (2007). BEAST: bayesian evolutionary analysis by sampling trees. BMC Evol. Biol. 7:214. 10.1186/1471-2148-7-214, PMID: 17996036 PMC2247476

[ref13] Edgar R. C. (2004). MUSCLE: multiple sequence alignment with high accuracy and high throughput. Nucleic Acids Res. 32, 1792–1797. 10.1093/nar/gkh340, PMID: 15034147 PMC390337

[ref14] Fang Y. Reisen W. K. (2006). Previous infection with West Nile or St. Louis encephalitis viruses provides cross protection during reinfection in house finches. Am. J. Trop. Med. Hyg. 75, 480–485. 10.4269/ajtmh.2006.75.480, PMID: 16968925

[ref55] Fitch W. M. (1971). Toward defining the course of evolution: minimum change for a specific tree topology. Syst. Zool. 20, 406–416. 10.2307/2412116

[ref15] Grubaugh N. D. Gangavarapu K. Quick J. Matteson N. L. Goes De Jesus J. Main B. J.. (2019). An amplicon-based sequencing framework for accurately measuring intrahost virus diversity using PrimalSeq and iVar. Genome Biol. 20:8. 10.1186/s13059-018-1618-7, PMID: 30621750 PMC6325816

[ref16] Hepp C. M. Cocking J. H. Valentine M. Young S. J. Damian D. Samuels-Crow K. E.. (2018). Phylogenetic analysis of West Nile virus in Maricopa County, Arizona: evidence for dynamic behavior of strains in two major lineages in the American southwest. PLoS One 13:e0205801. 10.1371/journal.pone.0205801, PMID: 30475820 PMC6261030

[ref17] Hoyos-López R. Soto S. U. Rúa-Uribe G. Gallego-Gómez J. C. (2015). Molecular identification of Saint Louis encephalitis virus genotype IV in Colombia. Mem. Inst. Oswaldo Cruz 110, 719–725. 10.1590/0074-02760280040, PMID: 26313538 PMC4667573

[ref18] Jones S. C. Morris J. Hill G. Alderman M. Ratard R. C. (2002). St. Louis encephalitis outbreak in Louisiana in 2001. J. La. State Med. Soc. 154, 303–306. PMID: 12517026

[ref19] Kalyaanamoorthy S. Minh B. Q. Wong T. K. F. von Haeseler A. Jermiin L. S. (2017). ModelFinder: fast model selection for accurate phylogenetic estimates. Nat. Methods 14, 587–589. 10.1038/nmeth.4285, PMID: 28481363 PMC5453245

[ref20] Kramer L. D. Chandler L. J. (2001). Phylogenetic analysis of the envelope gene of St. Louis encephalitis virus. Arch. Virol. 146, 2341–2355. 10.1007/s007050170007, PMID: 11811684

[ref21] Lanciotti R. S. Roehrig J. T. Deubel V. Smith J. Parker M. Steele K.. (1999). Origin of the West Nile virus responsible for an outbreak of encephalitis in the Northeastern United States. Science 286, 2333–2337. 10.1126/science.286.5448.2333, PMID: 10600742

[ref22] Langmead B. Salzberg S. L. (2012). Fast gapped-read alignment with bowtie 2. Nat. Methods 9, 357–359. 10.1038/nmeth.1923, PMID: 22388286 PMC3322381

[ref23] Li H. Handsaker B. Wysoker A. Fennell T. Ruan J. Homer N.. (2009). The sequence alignment/map format and SAMtools. Bioinformatics 25, 2078–2079. 10.1093/bioinformatics/btp352, PMID: 19505943 PMC2723002

[ref24] Lord R. D. Calisher C. H. Doughty W. P. (1974). Assessment of bird involvement in three urban St. Louis encephalitis epidemics. Am. J. Epidemiol. 99:367. 10.1093/oxfordjournals.aje.a121622, PMID: 4825601

[ref25] Maharaj P. D. Bosco-Lauth A. M. Langevin S. A. Anishchenko M. Bowen R. A. Reisen W. K.. (2018). West Nile and St. Louis encephalitis viral genetic determinants of avian host competence. PLoS Negl. Trop. Dis. 12:e0006302. 10.1371/journal.pntd.0006302, PMID: 29447156 PMC5831645

[ref26] Nguyen L.-T. Schmidt H. A. von Haeseler A. Minh B. Q. (2015). IQ-TREE: a fast and effective stochastic algorithm for estimating maximum-likelihood phylogenies. Mol. Biol. Evol. 32, 268–274. 10.1093/molbev/msu300, PMID: 25371430 PMC4271533

[ref27] Ottendorfer C. L. Ambrose J. H. White G. S. Unnasch T. R. Stark L. M. (2009). Isolation of genotype V St. Louis encephalitis virus in Florida. Emerg. Infect. Dis. 15, 604–606. 10.3201/eid1504.081094, PMID: 19331744 PMC2671428

[ref28] Parker J. Rambaut A. Pybus O. G. (2008). Correlating viral phenotypes with phylogeny: accounting for phylogenetic uncertainty. Infect. Genet. Evol. 8, 239–246. 10.1016/j.meegid.2007.08.001, PMID: 17921073

[ref29] Pedersen K. Marks D. R. Wang E. Eastwood G. Weaver S. C. Goldstein S. M.. (2016). Widespread detection of antibodies to eastern equine encephalitis, West Nile, St. Louis encephalitis, and Turlock viruses in various species of wild birds from across the United States. Am. J. Trop. Med. Hyg. 95, 206–211. 10.4269/ajtmh.15-0840, PMID: 27162269 PMC4944691

[ref30] Quick J. Grubaugh N. D. Pullan S. T. Claro I. M. Smith A. D. Gangavarapu K.. (2017). Multiplex PCR method for MinION and Illumina sequencing of zika and other virus genomes directly from clinical samples. Nat. Protoc. 12, 1261–1276. 10.1038/nprot.2017.066, PMID: 28538739 PMC5902022

[ref31] Rambaut A. Drummond A. J. Xie D. Baele G. Suchard M. A. (2018). Posterior summarization in bayesian phylogenetics using tracer 1.7. Syst. Biol. 67, 901–904. 10.1093/sysbio/syy032, PMID: 29718447 PMC6101584

[ref32] Rambaut A. Lam T. T. Carvalho L. M. Pybus O. G. (2016). Exploring the temporal structure of heterochronous sequences using TempEst (formerly path-O-gen). Virus Evol. 2:vew007. 10.1093/ve/vew007, PMID: 27774300 PMC4989882

[ref33] Reisen W. K. (2003). Epidemiology of St. Louis encephalitis virus. Adv. Virus Res. 61, 139–183. 10.1016/s0065-3527(03)61004-3, PMID: 14714432

[ref34] Reisen W. K. (2013). Ecology of West Nile virus in North America. Viruses 5, 2079–2105. 10.3390/v5092079, PMID: 24008376 PMC3798891

[ref35] Reisen W. K. Chiles R. E. Martinez V. M. Fang Y. Green E. N. (2003). Experimental infection of California birds with western equine encephalomyelitis and St. Louis encephalitis viruses. J. Med. Entomol. 40, 968–982. 10.1603/0022-2585-40.6.968, PMID: 14765678

[ref36] Reisen W. K. Fang Y. Lothrop H. D. Martinez V. M. Wilson J. Oconnor P.. (2006). Overwintering of West Nile virus in Southern California. J. Med. Entomol. 43, 344–355. 10.1093/jmedent/43.2.344, PMID: 16619621

[ref37] Reisen W. K. Fang Y. Martinez V. M. (2005). Avian host and mosquito (Diptera: Culicidae) vector competence determine the efficiency of West Nile and St. Louis encephalitis virus transmission. J. Med. Entomol. 42, 367–375. 10.1603/0022-2585(2005)042[0367:AHAMDC]2.0.CO;2, PMID: 15962789

[ref38] Reisen W. K. Hardy J. L. Lothrop H. D. (1995b). Landscape ecology of arboviruses in southern California: patterns in the epizootic dissemination of western equine encephalomyelitis and St. Louis encephalitis viruses in Coachella Valley, 1991–1992. J. Med. Entomol. 32, 267–275. 10.1093/jmedent/32.3.267, PMID: 7616516

[ref39] Reisen W. K. Lothrop H. D. Chiles R. E. Cusack R. Green E. G. N. Fang Y.. (2002). Persistence and amplification of St. Louis encephalitis virus in the Coachella Valley of California, 2000-2001. J. Med. Entomol. 39, 793–805. 10.1603/0022-2585-39.5.793, PMID: 12349864

[ref40] Reisen W. K. Lothrop H. D. Presser S. B. Milby M. M. Hardy J. L. Wargo M. J.. (1995a). Landscape ecology of arboviruses in southern California: temporal and spatial patterns of vector and virus activity in Coachella Valley, 1990–1992. J. Med. Entomol. 32, 255–266. 10.1093/jmedent/32.3.255, PMID: 7616515

[ref41] Reisen W. K. Lothrop H. D. Wheeler S. S. Kennsington M. Gutierrez A. Fang Y.. (2008). Persistent West Nile virus transmission and the apparent displacement St. Louis encephalitis virus in Southeastern California, 2003-2006. J. Med. Entomol. 45, 494–508. 10.1603/0022-2585(2008)45[494:pwnvta]2.0.co;2, PMID: 18533445 PMC2435167

[ref42] Reisen W. K. Wheeler S. S. (2016). Surveys for antibodies against mosquitoborne encephalitis viruses in California birds, 1996-2013. Vector Borne Zoonotic Dis. 16, 264–282. 10.1089/vbz.2015.1888, PMID: 26974395 PMC4800269

[ref43] Robinson J. T. Thorvaldsdóttir H. Wenger A. M. Zehir A. Mesirov J. P. (2017). Variant review with the integrative genomics viewer. Cancer Res. 77, e31–e34. 10.1158/0008-5472.CAN-17-0337, PMID: 29092934 PMC5678989

[ref44] Suchard M. A. Lemey P. Baele G. Ayres D. L. Drummond A. J. Rambaut A. (2018). Bayesian phylogenetic and phylodynamic data integration using BEAST 1.10. Virus Evol. 4:vey016. 10.1093/ve/vey016, PMID: 29942656 PMC6007674

[ref45] Swetnam D. M. Stuart J. B. Young K. Maharaj P. D. Fang Y. Garcia S.. (2020). Movement of St. Louis encephalitis virus in the Western United States, 2014-2018. PLoS Negl. Trop. Dis. 14:e0008343. 10.1371/journal.pntd.0008343, PMID: 32520944 PMC7307790

[ref46] Tesh R. B. Amelia P. A. da Rosa T. Guzman H. Araujo T. P. Xiao S. -Y. (2002). Immunization with heterologous flaviviruses protective against fatal West Nile encephalitis. Emerg. Infect. Dis. 8, 245–251. 10.3201/eid0803.010238, PMID: 11927020 PMC2732478

[ref47] Wang T. H. Donaldson Y. K. Brettle R. P. Bell J. E. Simmonds P. (2001). Identification of shared populations of human immunodeficiency virus type 1 infecting microglia and tissue macrophages outside the central nervous system. J. Virol. 75, 11686–11699. 10.1128/JVI.75.23.11686-11699.2001, PMID: 11689650 PMC114755

[ref48] Wang H.-C. Minh B. Q. Susko E. Roger A. J. (2018). Modeling site heterogeneity with posterior mean site frequency profiles accelerates accurate phylogenomic estimation. Syst. Biol. 67, 216–235. 10.1093/sysbio/syx068, PMID: 28950365

[ref49] Webster L. T. Fite G. L. (1933). A virus encountered in the study of material from cases of encephalitis n the St. Louis and Kansas City epidemics of 1933. Science 78, 463–465. 10.1126/science.78.2029.463, PMID: 17752093

[ref50] White G. S. Symmes K. Sun P. Fang Y. Garcia S. Steiner C.. (2016). Reemergence of St. Louis encephalitis virus, California, 2015. Emerg. Infect. Dis. 22, 2185–2188. 10.3201/eid2212.160805, PMID: 27869600 PMC5189155

[ref51] Whitfield J. B. Lockhart P. J. (2007). Deciphering ancient rapid radiations. Trends Ecol. Evol. 22, 258–265. 10.1016/j.tree.2007.01.012, PMID: 17300853

[ref52] Yu G. (2020). Using Ggtree to visualize data on tree-like structures. Curr. Protoc. Bioinformatics 69:e96. 10.1002/cpbi.96, PMID: 32162851

[ref53] Yu G. David K. S. Zhu H. Guan Y. Lam T. T.-Y. (2017). Ggtree: an R package for visualization and annotation of phylogenetic trees with their covariates and other associated data. Methods Ecol. Evol. 8, 28–36. 10.1111/2041-210x.12628

[ref54] Yu G. Lam T. T.-Y. Zhu H. Guan Y. (2018). Two methods for mapping and visualizing associated data on phylogeny using Ggtree. Mol. Biol. Evol. 35, 3041–3043. 10.1093/molbev/msy194, PMID: 30351396 PMC6278858

