# Supplementary material for: St. Louis Encephalitis Virus in the Southwestern United States: A Phylogeographic Case for a Multi-Variant Introduction Event
Source: Front Genet. 2021 Jun 8;12:667895. doi: 10.3389/fgene.2021.667895 (PMC8217752; doi:10.3389/fgene.2021.667895)
Supplement: Supplementary file 1 [file Data_Sheet_1.docx]

# Supplemental


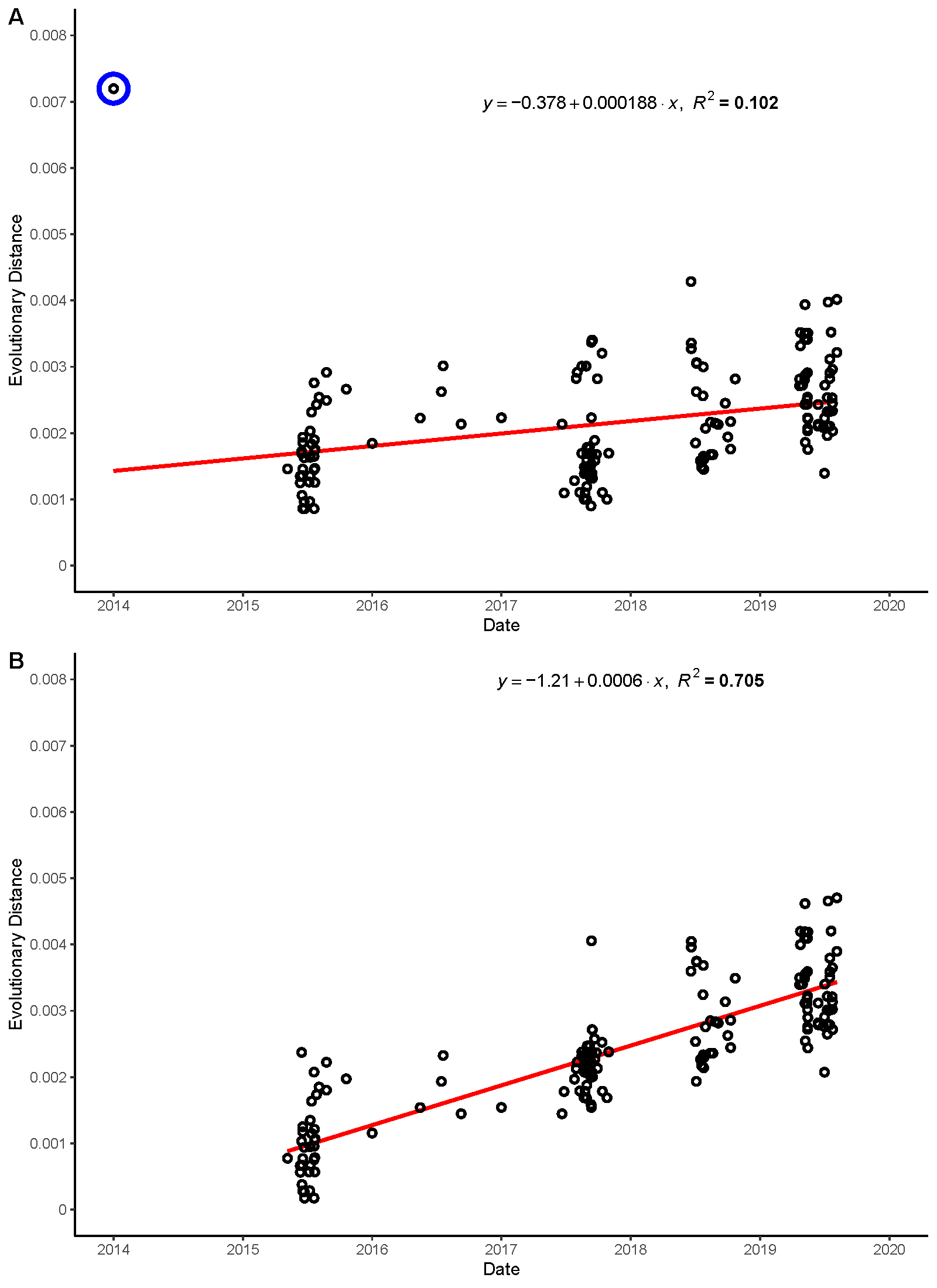


Figure S1: (A) Linear regression of evolutionary distance versus time for all 175 genomes. The 2014 SLEV sample (KX965720) from Maricopa County, highlighted in the blue circle, was removed from further analysis as an outlier due to it having an unexpectedly high evolutionary distance. (B) Linear regression of genetic distance versus time for all 174 genomes used to determine if a molecular signal of genetic change over time was detectable.

*A*

*
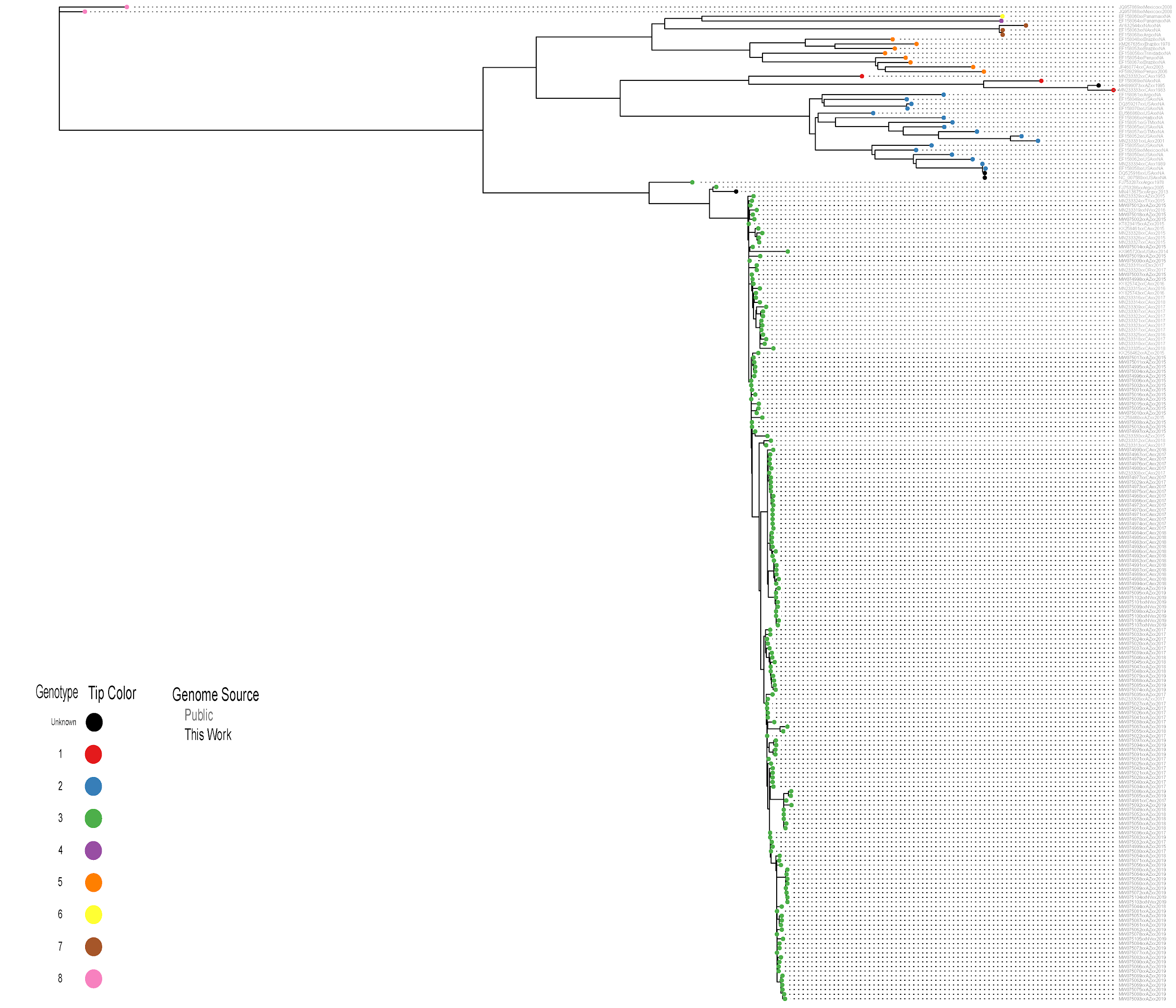
*

*B*

*
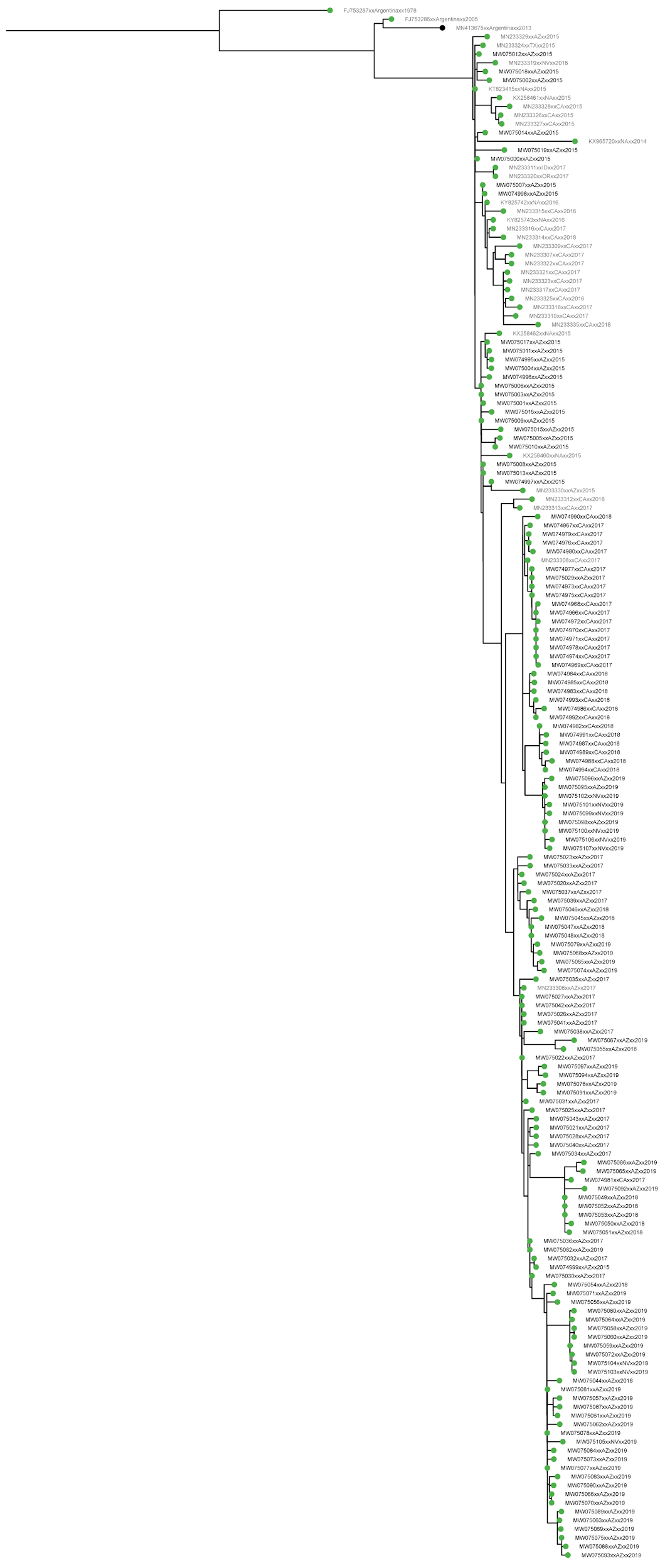
*

*Figure S2: (A)* The maximum likelihood phylogenetic tree reconstructed using 216 SLEV genomes. The colored tips indicate the genotype of the respective sample. The gray tip labels indicate publicly available samples while the black samples are samples sequenced by our lab. All the samples post-2015 outbreak sequenced by our lab are genotype III and nested within the Argentinian 2005 and 2010 samples. (B) Zoom in on genotype III clade formed under the 1978 Argentinian variant (FJ753287) which shows that all samples in the southwest since 2015 are of genotype III.


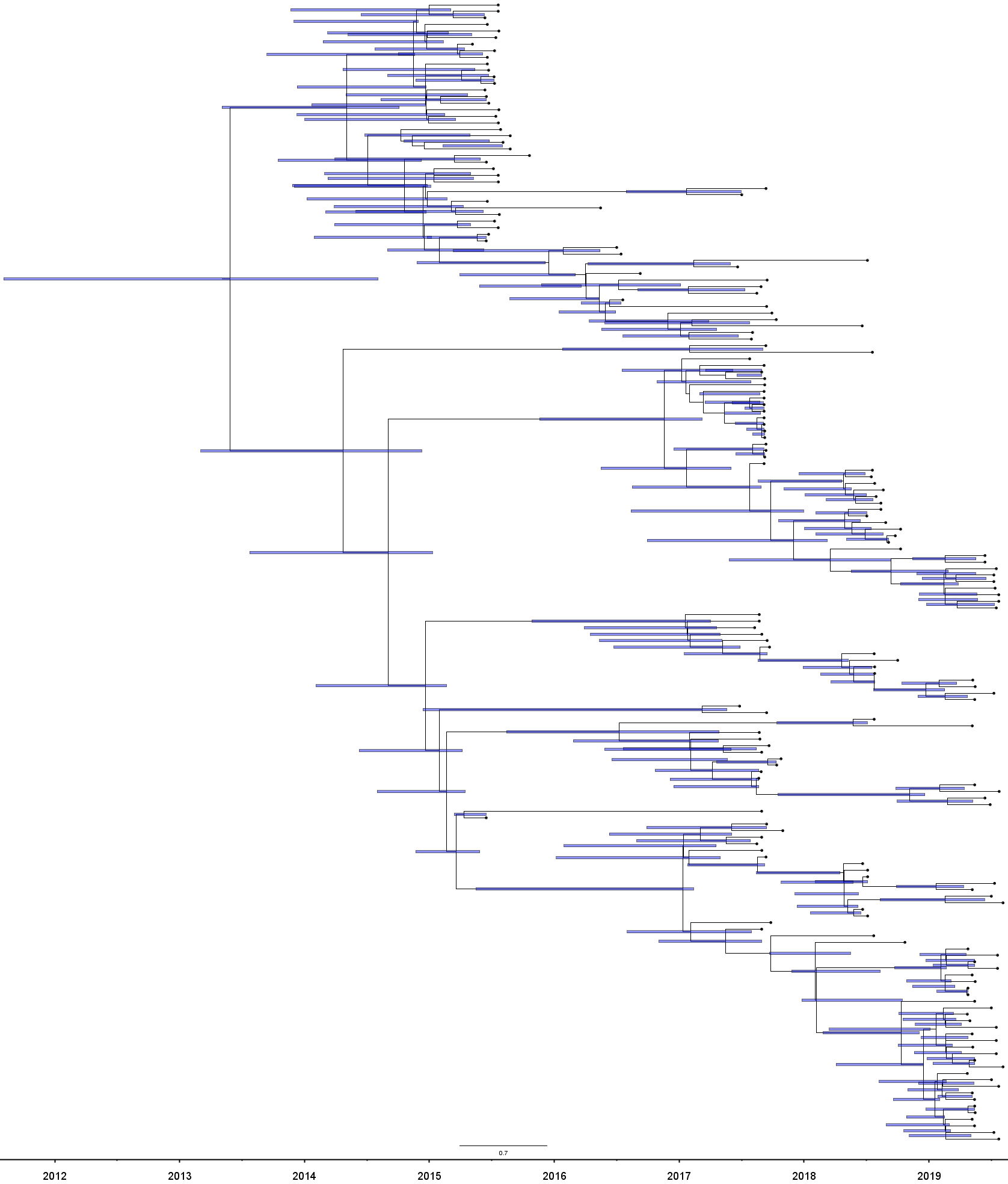


**Figure S3:The maximum clade credibility phylogenetic tree reconstructed using 174 genotype III SLEV genomes throughout the Southwest.** The node bars represent the 95% HPD Credible Intervals for the age estimates at each node.

Table S1: Meta Data for all samples used in the analyses.

| Accession Number | Collection Date | Source | | Country | | State | | County | Vector/Host | Genotype | | | Lat | Lon |
| --- | --- | --- | --- | --- | --- | --- | --- | --- | --- | --- | --- | --- | --- | --- |
| MW074967 | 9/6/2017 | | CVMVCD | | USA | CA | Riverside | | *Culex tarsalis* | | 3 | 33.533 | | -116.035 |
| MW074969 | 9/6/2017 | | CVMVCD | | USA | CA | Riverside | | *Culex tarsalis* | | 3 | 33.515 | | -115.935 |
| MW074970 | 9/6/2017 | | CVMVCD | | USA | CA | Riverside | | *Culex tarsalis* | | 3 | 33.521 | | -115.939 |
| MW074971 | 9/6/2017 | | CVMVCD | | USA | CA | Riverside | | *Culex tarsalis* | | 3 | 33.513 | | -115.931 |
| MW074972 | 9/6/2017 | | CVMVCD | | USA | CA | Riverside | | *Culex tarsalis* | | 3 | 33.533 | | -116.044 |
| MW074973 | 9/6/2017 | | CVMVCD | | USA | CA | Riverside | | *Culex tarsalis* | | 3 | 33.525 | | -116.026 |
| MW074974 | 9/8/2017 | | CVMVCD | | USA | CA | Riverside | | *Culex tarsalis* | | 3 | 33.533 | | -116.035 |
| MW074976 | 9/8/2017 | | CVMVCD | | USA | CA | Riverside | | *Culex tarsalis* | | 3 | 33.533 | | -116.035 |
| MW074977 | 9/8/2017 | | CVMVCD | | USA | CA | Riverside | | *Culex tarsalis* | | 3 | 33.533 | | -116.035 |
| MW074978 | 9/8/2017 | | CVMVCD | | USA | CA | Riverside | | *Culex tarsalis* | | 3 | 33.533 | | -116.035 |
| MW074979 | 9/12/2017 | | CVMVCD | | USA | CA | Riverside | | *Culex tarsalis* | | 3 | 33.525 | | -116.026 |
| MW074980 | 9/12/2017 | | CVMVCD | | USA | CA | Riverside | | *Culex tarsalis* | | 3 | 33.533 | | -116.044 |
| MW074981 | 9/12/2017 | | CVMVCD | | USA | CA | Riverside | | *Culex tarsalis* | | 3 | 33.533 | | -116.044 |
| MW074983 | 7/17/2018 | | CVMVCD | | USA | CA | Riverside | | *Culex tarsalis* | | 3 | 33.540 | | -116.062 |
| MW074984 | 7/20/2018 | | CVMVCD | | USA | CA | Riverside | | *Culex tarsalis* | | 3 | 33.533 | | -116.035 |
| MW074985 | 7/26/2018 | | CVMVCD | | USA | CA | Riverside | | *Culex tarsalis* | | 3 | 33.540 | | -116.079 |
| MW074986 | 7/31/2018 | | CVMVCD | | USA | CA | Riverside | | *Culex tarsalis* | | 3 | 33.581 | | -116.078 |
| MW074987 | 8/28/2018 | | CVMVCD | | USA | CA | Riverside | | *Culex tarsalis* | | 3 | 33.533 | | -116.035 |
| MW074988 | 9/25/2018 | | CVMVCD | | USA | CA | Riverside | | *Culex tarsalis* | | 3 | 33.532 | | -116.031 |
| MW074990 | 10/10/2018 | | CVMVCD | | USA | CA | Riverside | | *Culex tarsalis* | | 3 | 33.532 | | -116.061 |
| MW074991 | 8/14/2018 | | CVMVCD | | USA | CA | Riverside | | *Culex tarsalis* | | 3 | 33.525 | | -115.973 |
| MW074992 | 8/14/2018 | | CVMVCD | | USA | CA | Riverside | | *Culex tarsalis* | | 3 | 33.525 | | -115.957 |
| MW074993 | 8/21/2018 | | CVMVCD | | USA | CA | Riverside | | *Culex tarsalis* | | 3 | 33.525 | | -116.096 |
| MW074994 | 9/5/2018 | | CVMVCD | | USA | CA | Riverside | | *Culex tarsalis* | | 3 | 33.540 | | -116.079 |
| MW074996 | 6/12/2015 | | MCESVCD | | USA | AZ | Maricopa | | *Culex quinquefasciatus* | | 3 | 33.389 | | -111.850 |
| MW074997 | 6/12/2015 | | MCESVCD | | USA | AZ | Maricopa | | *Culex quinquefasciatus* | | 3 | 33.388 | | -111.827 |
| MW074999 | 6/16/2015 | | MCESVCD | | USA | AZ | Maricopa | | *Culex quinquefasciatus* | | 3 | 33.431 | | -111.851 |
| MW075000 | 6/17/2015 | | MCESVCD | | USA | AZ | Maricopa | | *Culex quinquefasciatus* | | 3 | 33.547 | | -112.159 |
| MW075001 | 6/17/2015 | | MCESVCD | | USA | AZ | Maricopa | | *Culex quinquefasciatus* | | 3 | 33.241 | | -111.781 |
| MW075002 | 6/19/2015 | | MCESVCD | | USA | AZ | Maricopa | | *Culex quinquefasciatus* | | 3 | 33.359 | | -111.837 |
| MW075004 | 6/19/2015 | | MCESVCD | | USA | AZ | Maricopa | | *Culex quinquefasciatus* | | 3 | 33.444 | | -111.708 |
| MW075005 | 6/19/2015 | | MCESVCD | | USA | AZ | Maricopa | | *Culex quinquefasciatus* | | 3 | 33.449 | | -111.692 |
| MW075006 | 6/23/2015 | | MCESVCD | | USA | AZ | Maricopa | | *Culex quinquefasciatus* | | 3 | 33.430 | | -112.247 |
| MW075008 | 6/24/2015 | | MCESVCD | | USA | AZ | Maricopa | | *Culex quinquefasciatus* | | 3 | 33.361 | | -111.759 |
| MW075012 | 7/21/2015 | | MCESVCD | | USA | AZ | Maricopa | | *Culex quinquefasciatus* | | 3 | 33.290 | | -111.764 |
| MW075013 | 7/21/2015 | | MCESVCD | | USA | AZ | Maricopa | | *Culex quinquefasciatus* | | 3 | 33.297 | | -111.769 |
| MW075014 | 7/22/2015 | | MCESVCD | | USA | AZ | Maricopa | | *Culex quinquefasciatus* | | 3 | 33.241 | | -111.781 |
| MW075016 | 7/23/2015 | | MCESVCD | | USA | AZ | Maricopa | | *Culex quinquefasciatus* | | 3 | 33.662 | | -112.126 |
| MW075017 | 7/23/2015 | | MCESVCD | | USA | AZ | Maricopa | | *Culex quinquefasciatus* | | 3 | 33.358 | | -111.730 |
| MW075018 | 7/24/2015 | | MCESVCD | | USA | AZ | Maricopa | | *Culex quinquefasciatus* | | 3 | 33.387 | | -111.793 |
| MW075019 | 10/20/2015 | | MCESVCD | | USA | AZ | Maricopa | | *Culex quinquefasciatus* | | 3 | 33.476 | | -111.988 |
| MW075032 | 8/30/2017 | | MCESVCD | | USA | AZ | Maricopa | | *Culex quinquefasciatus* | | 3 | 33.384 | | -111.766 |
| MW075046 | 7/25/2018 | | MCESVCD | | USA | AZ | Maricopa | | *Culex quinquefasciatus* | | 3 | 33.298 | | -111.778 |
| MW075047 | 7/26/2018 | | MCESVCD | | USA | AZ | Maricopa | | *Culex quinquefasciatus* | | 3 | 33.269 | | -111.710 |
| MW075048 | 7/26/2018 | | MCESVCD | | USA | AZ | Maricopa | | *Culex quinquefasciatus* | | 3 | 33.255 | | -111.716 |
| MW075049 | 7/6/2018 | | MCESVCD | | USA | AZ | Maricopa | | *Culex quinquefasciatus* | | 3 | 33.343 | | -111.764 |
| MW075052 | 7/6/2018 | | MCESVCD | | USA | AZ | Maricopa | | *Culex tarsalis* | | 3 | 33.328 | | -111.698 |
| MW075053 | 7/6/2018 | | MCESVCD | | USA | AZ | Maricopa | | *Culex tarsalis* | | 3 | 33.358 | | -111.730 |
| MW075055 | 7/25/2018 | | MCESVCD | | USA | AZ | Maricopa | | *Culex tarsalis* | | 3 | 33.618 | | -112.142 |
| MW075060 | 4/25/2019 | | MCESVCD | | USA | AZ | Maricopa | | *Culex quinquefasciatus* | | 3 | 33.472 | | -112.211 |
| MW075063 | 5/7/2019 | | MCESVCD | | USA | AZ | Maricopa | | *Culex tarsalis* | | 3 | 33.394 | | -112.254 |
| MW075066 | 5/8/2019 | | MCESVCD | | USA | AZ | Maricopa | | *Culex quinquefasciatus* | | 3 | 33.412 | | -112.246 |
| MW075071 | 5/14/2019 | | MCESVCD | | USA | AZ | Maricopa | | *Culex quinquefasciatus* | | 3 | 33.389 | | -111.780 |
| MW075073 | 5/15/2019 | | MCESVCD | | USA | AZ | Maricopa | | *Culex tarsalis* | | 3 | 33.401 | | -112.212 |
| MW075076 | 5/15/2019 | | MCESVCD | | USA | AZ | Maricopa | | *Culex quinquefasciatus* | | 3 | 33.289 | | -111.766 |
| MW075079 | 5/16/2019 | | MCESVCD | | USA | AZ | Maricopa | | *Culex quinquefasciatus* | | 3 | 33.591 | | -112.129 |
| MW075085 | 7/10/2019 | | MCESVCD | | USA | AZ | Maricopa | | *Culex tarsalis* | | 3 | 33.643 | | -112.196 |
| MW075088 | 7/17/2019 | | MCESVCD | | USA | AZ | Maricopa | | *Culex tarsalis* | | 3 | 33.551 | | -112.415 |
| MW075089 | 7/17/2019 | | MCESVCD | | USA | AZ | Maricopa | | *Culex quinquefasciatus* | | 3 | 33.341 | | -111.883 |
| MW075093 | 8/6/2019 | | MCESVCD | | USA | AZ | Maricopa | | *Culex quinquefasciatus* | | 3 | 33.571 | | -112.454 |
| MW075099 | 7/11/2019 | | SNHD | | USA | NV | Clark | | *Culex tarsalis* | | 3 | 36.210 | | -115.174 |
| MW075100 | 7/11/2019 | | SNHD | | USA | NV | Clark | | *Culex stigmatasoma* | | 3 | 36.204 | | -115.141 |
| MW075101 | 7/17/2019 | | SNHD | | USA | NV | Clark | | *Culex quinquefasciatus* | | 3 | 36.178 | | -115.187 |
| MW075102 | 7/17/2019 | | SNHD | | USA | NV | Clark | | *Culex quinquefasciatus* | | 3 | 36.148 | | -115.311 |
| MW075103 | 7/22/2019 | | SNHD | | USA | NV | Clark | | *Culex quinquefasciatus* | | 3 | 36.146 | | -115.303 |
| MW075104 | 7/23/2019 | | SNHD | | USA | NV | Clark | | *Culex quinquefasciatus* | | 3 | 36.193 | | -115.288 |
| MW075105 | 7/24/2019 | | SNHD | | USA | NV | Clark | | *Culex quinquefasciatus* | | 3 | 36.305 | | -115.261 |
| MW075106 | 7/25/2019 | | SNHD | | USA | NV | Clark | | *Culex quinquefasciatus* | | 3 | 36.180 | | -115.198 |
| MW075107 | 7/25/2019 | | SNHD | | USA | NV | Clark | | *Culex quinquefasciatus* | | 3 | 36.097 | | -115.268 |
| MW074966 | 9/6/2017 | | CVMVCD | | USA | CA | Riverside | | *Culex tarsalis* | | 3 | 33.533 | | -116.035 |
| MW074968 | 9/6/2017 | | CVMVCD | | USA | CA | Riverside | | *Culex tarsalis* | | 3 | 33.533 | | -116.035 |
| MW074975 | 9/8/2017 | | CVMVCD | | USA | CA | Riverside | | *Culex tarsalis* | | 3 | 33.533 | | -116.035 |
| MW074982 | 7/3/2018 | | CVMVCD | | USA | CA | Riverside | | *Culex tarsalis* | | 3 | 33.532 | | -116.061 |
| MW074989 | 10/10/2018 | | CVMVCD | | USA | CA | Riverside | | *Culex tarsalis* | | 3 | 33.525 | | -116.026 |
| MW074995 | 5/7/2015 | | MCESVCD | | USA | AZ | Maricopa | | *Culex quinquefasciatus* | | 3 | 33.359 | | -111.667 |
| MW074998 | 6/16/2015 | | MCESVCD | | USA | AZ | Maricopa | | *Culex tarsalis* | | 3 | 33.430 | | -112.247 |
| MW075003 | 6/19/2015 | | MCESVCD | | USA | AZ | Maricopa | | *Culex tarsalis* | | 3 | 33.325 | | -111.696 |
| MW075007 | 6/23/2015 | | MCESVCD | | USA | AZ | Maricopa | | *Culex quinquefasciatus* | | 3 | 33.430 | | -112.247 |
| MW075009 | 7/9/2015 | | MCESVCD | | USA | AZ | Maricopa | | *Culex quinquefasciatus* | | 3 | 33.430 | | -111.729 |
| MW075011 | 7/10/2015 | | MCESVCD | | USA | AZ | Maricopa | | *Culex quinquefasciatus* | | 3 | 33.366 | | -111.649 |
| MW075015 | 7/22/2015 | | MCESVCD | | USA | AZ | Maricopa | | *Culex quinquefasciatus* | | 3 | 33.329 | | -111.815 |
| MW075020 | 8/10/2017 | | MCESVCD | | USA | AZ | Maricopa | | *Culex tarsalis* | | 3 | 33.585 | | -112.209 |
| MW075021 | 8/16/2017 | | MCESVCD | | USA | AZ | Maricopa | | *Culex quinquefasciatus* | | 3 | 33.557 | | -112.194 |
| MW075022 | 8/22/2017 | | MCESVCD | | USA | AZ | Maricopa | | *Culex quinquefasciatus* | | 3 | 33.573 | | -112.435 |
| MW075023 | 8/23/2017 | | MCESVCD | | USA | AZ | Maricopa | | *Culex quinquefasciatus* | | 3 | 33.634 | | -111.987 |
| MW075024 | 8/23/2017 | | MCESVCD | | USA | AZ | Maricopa | | *Culex quinquefasciatus* | | 3 | 33.387 | | -111.793 |
| MW075025 | 8/23/2017 | | MCESVCD | | USA | AZ | Maricopa | | *Culex quinquefasciatus* | | 3 | 33.474 | | -111.968 |
| MW075026 | 8/25/2017 | | MCESVCD | | USA | AZ | Maricopa | | *Culex quinquefasciatus* | | 3 | 33.430 | | -112.399 |
| MW075027 | 8/29/2017 | | MCESVCD | | USA | AZ | Maricopa | | *Culex quinquefasciatus* | | 3 | 33.473 | | -112.039 |
| MW075028 | 8/30/2017 | | MCESVCD | | USA | AZ | Maricopa | | *Culex tarsalis* | | 3 | 33.547 | | -112.109 |
| MW075030 | 8/30/2017 | | MCESVCD | | USA | AZ | Maricopa | | *Culex quinquefasciatus* | | 3 | 33.633 | | -112.005 |
| MW075033 | 8/31/2017 | | MCESVCD | | USA | AZ | Maricopa | | *Culex tarsalis* | | 3 | 33.469 | | -112.297 |
| MW075034 | 8/31/2017 | | MCESVCD | | USA | AZ | Maricopa | | *Culex quinquefasciatus* | | 3 | 33.315 | | -111.747 |
| MW075035 | 9/14/2017 | | MCESVCD | | USA | AZ | Maricopa | | *Culex tarsalis* | | 3 | 33.227 | | -111.729 |
| MW075036 | 9/14/2017 | | MCESVCD | | USA | AZ | Maricopa | | *Culex tarsalis* | | 3 | 33.328 | | -111.763 |
| MW075037 | 9/15/2017 | | MCESVCD | | USA | AZ | Maricopa | | *Culex tarsalis* | | 3 | 33.317 | | -111.715 |
| MW075038 | 9/21/2017 | | MCESVCD | | USA | AZ | Maricopa | | *Culex quinquefasciatus* | | 3 | 33.585 | | -112.161 |
| MW075039 | 9/22/2017 | | MCESVCD | | USA | AZ | Maricopa | | *Culex tarsalis* | | 3 | 33.344 | | -111.712 |
| MW075040 | 9/26/2017 | | MCESVCD | | USA | AZ | Maricopa | | *Culex quinquefasciatus* | | 3 | 33.391 | | -112.293 |
| MW075041 | 10/13/2017 | | MCESVCD | | USA | AZ | Maricopa | | *Culex quinquefasciatus* | | 3 | 33.616 | | -112.162 |
| MW075042 | 10/26/2017 | | MCESVCD | | USA | AZ | Maricopa | | *Culex tarsalis* | | 3 | 33.328 | | -111.763 |
| MW075043 | 10/31/2017 | | MCESVCD | | USA | AZ | Maricopa | | *Culex quinquefasciatus* | | 3 | 33.411 | | -111.715 |
| MW075044 | 10/23/2018 | | MCESVCD | | USA | AZ | Maricopa | | *Culex quinquefasciatus* | | 3 | 33.373 | | -111.888 |
| MW075045 | 10/2/2018 | | MCESVCD | | USA | AZ | Maricopa | | *Culex quinquefasciatus* | | 3 | 33.273 | | -111.826 |
| MW075050 | 6/21/2018 | | MCESVCD | | USA | AZ | Maricopa | | *Culex tarsalis* | | 3 | 33.362 | | -111.732 |
| MW075054 | 7/24/2018 | | MCESVCD | | USA | AZ | Maricopa | | *Culex tarsalis* | | 3 | 33.357 | | -111.815 |
| MW075056 | 4/23/2019 | | MCESVCD | | USA | AZ | Maricopa | | *Culex tarsalis* | | 3 | 33.392 | | -112.343 |
| MW075057 | 4/23/2019 | | MCESVCD | | USA | AZ | Maricopa | | *Culex quinquefasciatus* | | 3 | 33.451 | | -111.950 |
| MW075058 | 4/25/2019 | | MCESVCD | | USA | AZ | Maricopa | | *Culex tarsalis* | | 3 | 33.472 | | -112.211 |
| MW075059 | 4/25/2019 | | MCESVCD | | USA | AZ | Maricopa | | *Culex quinquefasciatus* | | 3 | 33.484 | | -112.251 |
| MW075061 | 5/1/2019 | | MCESVCD | | USA | AZ | Maricopa | | *Culex tarsalis* | | 3 | 33.441 | | -111.873 |
| MW075062 | 5/7/2019 | | MCESVCD | | USA | AZ | Maricopa | | *Culex tarsalis* | | 3 | 33.396 | | -112.315 |
| MW075065 | 5/8/2019 | | MCESVCD | | USA | AZ | Maricopa | | *Culex quinquefasciatus* | | 3 | 33.429 | | -112.246 |
| MW075068 | 5/9/2019 | | MCESVCD | | USA | AZ | Maricopa | | *Culex tarsalis* | | 3 | 33.474 | | -112.246 |
| MW075069 | 5/9/2019 | | MCESVCD | | USA | AZ | Maricopa | | *Culex quinquefasciatus* | | 3 | 33.663 | | -112.299 |
| MW075070 | 5/14/2019 | | MCESVCD | | USA | AZ | Maricopa | | *Culex quinquefasciatus* | | 3 | 33.400 | | -112.274 |
| MW075072 | 5/15/2019 | | MCESVCD | | USA | AZ | Maricopa | | *Culex tarsalis* | | 3 | 33.384 | | -112.213 |
| MW075078 | 5/16/2019 | | MCESVCD | | USA | AZ | Maricopa | | *Culex quinquefasciatus* | | 3 | 33.484 | | -112.251 |
| MW075081 | 7/2/2019 | | MCESVCD | | USA | AZ | Maricopa | | *Culex tarsalis* | | 3 | 33.440 | | -111.889 |
| MW075082 | 7/2/2019 | | MCESVCD | | USA | AZ | Maricopa | | *Culex quinquefasciatus* | | 3 | 33.445 | | -111.761 |
| MW075083 | 7/3/2019 | | MCESVCD | | USA | AZ | Maricopa | | *Culex tarsalis* | | 3 | 33.430 | | -112.399 |
| MW075084 | 7/10/2019 | | MCESVCD | | USA | AZ | Maricopa | | *Culex tarsalis* | | 3 | 33.502 | | -112.017 |
| MW075086 | 7/12/2019 | | MCESVCD | | USA | AZ | Maricopa | | *Culex quinquefasciatus* | | 3 | 33.575 | | -112.216 |
| MW075087 | 7/17/2019 | | MCESVCD | | USA | AZ | Maricopa | | *Culex tarsalis* | | 3 | 33.441 | | -111.873 |
| MW075090 | 7/24/2019 | | MCESVCD | | USA | AZ | Maricopa | | *Culex quinquefasciatus* | | 3 | 33.440 | | -112.403 |
| MW075091 | 7/25/2019 | | MCESVCD | | USA | AZ | Maricopa | | *Culex tarsalis* | | 3 | 33.374 | | -111.709 |
| MW075092 | 8/6/2019 | | MCESVCD | | USA | AZ | Maricopa | | *Culex quinquefasciatus* | | 3 | 33.434 | | -111.730 |
| MW075094 | 6/18/2019 | | YCPA | | USA | AZ | Yuma | | *Culex tarsalis* | | 3 | 32.709 | | -114.612 |
| MW075096 | 6/24/2019 | | YCPA | | USA | AZ | Yuma | | *Culex tarsalis* | | 3 | 32.720 | | -114.599 |
| MW075098 | 7/15/2019 | | YCPA | | USA | AZ | Yuma | | *Culex tarsalis* | | 3 | 32.719 | | -114.610 |
| MW075010 | 7/10/2015 | | MCESVCD | | USA | AZ | Maricopa | | *Culex quinquefasciatus* | | 3 | 33.633 | | -112.070 |
| MW075029 | 8/30/2017 | | MCESVCD | | USA | AZ | Maricopa | | *Culex quinquefasciatus* | | 3 | 33.558 | | -112.440 |
| MW075031 | 8/30/2017 | | MCESVCD | | USA | AZ | Maricopa | | *Culex tarsalis* | | 3 | 33.404 | | -111.837 |
| MW075051 | 6/21/2018 | | MCESVCD | | USA | AZ | Maricopa | | *Culex tarsalis* | | 3 | 33.362 | | -111.732 |
| MW075064 | 5/7/2019 | | MCESVCD | | USA | AZ | Maricopa | | *Culex tarsalis* | | 3 | 33.395 | | -112.534 |
| MW075067 | 5/8/2019 | | MCESVCD | | USA | AZ | Maricopa | | *Culex quinquefasciatus* | | 3 | 33.636 | | -112.139 |
| MW075074 | 5/15/2019 | | MCESVCD | | USA | AZ | Maricopa | | *Culex tarsalis* | | 3 | 33.546 | | -112.144 |
| MW075075 | 5/15/2019 | | MCESVCD | | USA | AZ | Maricopa | | *Culex quinquefasciatus* | | 3 | 33.530 | | -112.431 |
| MW075077 | 5/15/2019 | | MCESVCD | | USA | AZ | Maricopa | | *Culex quinquefasciatus* | | 3 | 33.438 | | -112.344 |
| MW075080 | 5/16/2019 | | MCESVCD | | USA | AZ | Maricopa | | *Culex quinquefasciatus* | | 3 | 33.472 | | -112.211 |
| MW075095 | 6/18/2019 | | YCPA | | USA | AZ | Yuma | | *Culex tarsalis* | | 3 | 32.720 | | -114.599 |
| MW075097 | 7/1/2019 | | YCPA | | USA | AZ | Yuma | | *Culex tarsalis* | | 3 | 32.732 | | -114.656 |
| MN233335 | 6/20/2018 | | PUBLIC | | USA | CA | Tulare County | | *Culex quinquefasciatus* | | 3 | 36.220 | | -119.340 |
| MN233334 | 1/1/1989 | | PUBLIC | | USA | CA | Kern County | | *Culex tarsalis* | | 2 | NA | | NA |
| MN233333 | 1/1/1983 | | PUBLIC | | USA | CA | Coachella Valley | | *Culex tarsalis* | | 1 | NA | | NA |
| MN233332 | 1/1/1953 | | PUBLIC | | USA | CA | Kern County | | *Culex tarsalis* | | 1 | NA | | NA |
| MN233331 | 8/30/2001 | | PUBLIC | | USA | LA | Ouachita Parish | | *Culex quinquefasciatus* | | 2 | NA | | NA |
| MN233330 | 7/21/2015 | | PUBLIC | | USA | AZ | Phoenix | | *Culex quinquefasciatus* | | 3 | 33.440 | | -111.780 |
| MN233329 | 7/10/2015 | | PUBLIC | | USA | AZ | Phoenix | | *Culex quinquefasciatus* | | 3 | 33.440 | | -111.710 |
| MN233328 | 8/25/2015 | | PUBLIC | | USA | CA | Butte County | | *Culex tarsalis* | | 3 | 33.530 | | -115.960 |
| MN233327 | 8/4/2015 | | PUBLIC | | USA | CA | Butte County | | *Culex tarsalis* | | 3 | 33.530 | | -116.040 |
| MN233326 | 8/25/2015 | | PUBLIC | | USA | CA | Butte County | | *Culex tarsalis* | | 3 | 33.530 | | -116.040 |
| MN233325 | 7/20/2016 | | PUBLIC | | USA | CA | Kern County | | *Culex tarsalis* | | 3 | 35.070 | | -119.240 |
| MN233324 | 7/21/2015 | | PUBLIC | | USA | TX | El Paso | | *Culex quinquefasciatus* | | 3 | NA | | NA |
| MN233323 | 8/3/2017 | | PUBLIC | | USA | CA | Turlock | | *Culex quinquefasciatus* | | 3 | 37.520 | | -120.830 |
| MN233322 | 8/16/2017 | | PUBLIC | | USA | CA | Tulare County | | *Culex quinquefasciatus* | | 3 | 36.230 | | -119.360 |
| MN233321 | 7/31/2017 | | PUBLIC | | USA | CA | Sutter/Yuba County | | *Culex tarsalis* | | 3 | 38.980 | | -121.550 |
| MN233320 | 1/1/2017 | | PUBLIC | | USA | OR | Malheur County | | *Culex species* | | 3 | NA | | NA |
| MN233319 | 5/16/2016 | | PUBLIC | | USA | NV | Clark County | | *Culex tarsalis* | | 3 | 36.070 | | -115.110 |
| MN233318 | 9/14/2017 | | PUBLIC | | USA | CA | Merced County | | *Culex tarsalis* | | 3 | 37.310 | | -120.380 |
| MN233317 | 9/29/2017 | | PUBLIC | | USA | CA | Madera County | | *Culex quinquefasciatus* | | 3 | 36.930 | | -120.050 |
| MN233316 | 6/21/2017 | | PUBLIC | | USA | CA | Kern County | | *Culex quinquefasciatus* | | 3 | 35.380 | | -119.040 |
| MN233315 | 7/15/2016 | | PUBLIC | | USA | CA | Kern County | | *Culex quinquefasciatus* | | 3 | 35.190 | | -118.970 |
| MN233314 | 7/5/2018 | | PUBLIC | | USA | CA | Kern County | | *Culex quinquefasciatus* | | 3 | 35.340 | | -119.070 |
| MN233313 | 9/11/2017 | | PUBLIC | | USA | CA | Imperial County | | *Culex tarsalis* | | 3 | 32.830 | | -115.560 |
| MN233312 | 7/20/2018 | | PUBLIC | | USA | CA | Imperial County | | *Culex quinquefasciatus* | | 3 | 32.830 | | -115.560 |
| MN233311 | 9/12/2017 | | PUBLIC | | USA | ID | Gem County | | *Culex tarsalis* | | 3 | 43.520 | | -116.310 |
| MN233310 | 10/12/2017 | | PUBLIC | | USA | CA | Fresno | | *Culex tarsalis* | | 3 | 36.800 | | -120.380 |
| MN233309 | 9/15/2017 | | PUBLIC | | USA | CA | Delano | | *Culex quinquefasciatus* | | 3 | 35.690 | | -119.260 |
| MN233308 | 7/26/2017 | | PUBLIC | | USA | CA | Coachella Valley | | *Culex tarsalis* | | 3 | 33.530 | | -116.040 |
| MN233307 | 8/28/2017 | | PUBLIC | | USA | CA | Butte County | | *Culex tarsalis* | | 3 | 39.360 | | -121.580 |
| MN233306 | 6/27/2017 | | PUBLIC | | USA | AZ | Phoenix | | *Culex tarsalis* | | 3 | 33.390 | | -112.270 |
| MN413675 | 3/25/2013 | | PUBLIC | | Argentina | NA | NA | | Homo *sapiens* | | NA | NA | | NA |
| NC_007580 | NA | | PUBLIC | | USA | NA | NA | | *Homo sapiens* | | NA | NA | | NA |
| MH899073 | 1/1/1995 | | PUBLIC | | USA | AZ | NA | | mosquito | | NA | NA | | NA |
| EF158061 | NA | | PUBLIC | | Argentina | NA | NA | | Culex species | | 2 | NA | | NA |
| EF158070 | NA | | PUBLIC | | USA | NA | NA | | Culex species | | 2 | NA | | NA |
| EF158069 | NA | | PUBLIC | | USA | NA | NA | | Culex species | | 1 | NA | | NA |
| EF158068 | NA | | PUBLIC | | Argentina | NA | NA | | Culex species | | 7 | NA | | NA |
| EF158067 | NA | | PUBLIC | | Brazil | NA | NA | | Culex species | | 5 | NA | | NA |
| EF158066 | NA | | PUBLIC | | Haiti | NA | NA | | Culex species | | 2 | NA | | NA |
| EF158065 | NA | | PUBLIC | | USA | NA | NA | | Culex species | | 2 | NA | | NA |
| EF158064 | NA | | PUBLIC | | Panama | NA | NA | | Culex species | | 4 | NA | | NA |
| EF158063 | NA | | PUBLIC | | USA | NA | NA | | Culex species | | 7 | NA | | NA |
| EF158062 | NA | | PUBLIC | | USA | NA | NA | | Culex species | | 2 | NA | | NA |
| EF158060 | NA | | PUBLIC | | Panama | NA | NA | | Culex species | | 6 | NA | | NA |
| EF158059 | NA | | PUBLIC | | Mexico | NA | NA | | Culex species | | 2 | NA | | NA |
| EF158058 | NA | | PUBLIC | | USA | NA | NA | | Culex species | | 2 | NA | | NA |
| EF158057 | NA | | PUBLIC | | Guatemala | NA | NA | | Culex species | | 2 | NA | | NA |
| EF158056 | NA | | PUBLIC | | Trinidad | Trinidad | NA | | Culex species | | 5 | NA | | NA |
| EF158055 | NA | | PUBLIC | | USA | NA | NA | | Culex species | | 2 | NA | | NA |
| EF158054 | NA | | PUBLIC | | Peru | NA | NA | | Culex species | | 5 | NA | | NA |
| EF158053 | NA | | PUBLIC | | Brazil | NA | NA | | Culex species | | 5 | NA | | NA |
| EF158052 | NA | | PUBLIC | | USA | NA | NA | | Culex species | | 2 | NA | | NA |
| EF158051 | NA | | PUBLIC | | Guatemala | NA | NA | | Culex species | | 2 | NA | | NA |
| EF158050 | NA | | PUBLIC | | USA | NA | NA | | Culex species | | 2 | NA | | NA |
| EF158049 | NA | | PUBLIC | | USA | NA | NA | | Culex species | | 2 | NA | | NA |
| EF158048 | NA | | PUBLIC | | Brazil | NA | NA | | Culex species | | 5 | NA | | NA |
| KM267635 | 1/1/1978 | | PUBLIC | | Brazil | Belem | NA | | Homo *sapiens* | | 5 | NA | | NA |
| JF460774 | 1/1/2003 | | PUBLIC | | USA | CA | Imperial Valley | | *Culex tarsalis* | | 5 | NA | | NA |
| KF589299 | 3/27/2006 | | PUBLIC | | Peru | NA | NA | | Homo *sapiens* | | 5 | NA | | NA |
| FJ753287 | 1/1/1978 | | PUBLIC | | Argentina | NA | NA | | Culex species | | 3 | NA | | NA |
| FJ753286 | 1/1/2005 | | PUBLIC | | Argentina | NA | NA | | *Culex quinquefasciatus* | | 3 | NA | | NA |
| JQ957869 | 1/1/2008 | | PUBLIC | | Mexico | NA | NA | | *Culex nigripalpus* | | 8 | NA | | NA |
| JQ957868 | 1/1/2008 | | PUBLIC | | Mexico | NA | NA | | *Culex nigripalpus* | | 8 | NA | | NA |
| DQ525916 | NA | | PUBLIC | | USA | NA | NA | | *Culex nigripalpus* | | NA | NA | | NA |
| EU566860 | NA | | PUBLIC | | USA | NA | NA | | Homo *sapiens* | | 2 | NA | | NA |
| DQ359217 | NA | | PUBLIC | | USA | NA | NA | | *Homo sapiens* | | 2 | NA | | NA |
| AY632544 | NA | | PUBLIC | | USA | NA | NA | | *Homo sapiens* | | 7 | NA | | NA |
| KY825743 | 9/9/2016 | | PUBLIC | | USA | CA | NA | | *Homo sapiens* | | 3 | NA | | NA |
| KY825742 | 1/1/2016 | | PUBLIC | | USA | CA | NA | | *Culex pipiens* | | 3 | NA | | NA |
| KX258461 | 7/28/2015 | | PUBLIC | | USA | CA | Riverside | | *Culex pipiens* | | 3 | NA | | NA |
| KX258462 | 7/14/2015 | | PUBLIC | | USA | AZ | Maricopa | | *Culex pipiens* | | 3 | NA | | NA |
| KX258460 | 7/14/2015 | | PUBLIC | | USA | AZ | Maricopa | | *Culex tarsalis* | | 3 | NA | | NA |
| KX965720 | 1/1/2014 | | PUBLIC | | USA | AZ | Maricopa | | Culex species | | 3 | NA | | NA |
| KT823415 | 7/7/2015 | | PUBLIC | | USA | AZ | Maricopa | | *Culex quinquefasciatus* | | 3 | NA | | NA |

| Table S2: SLEV Multiplex PCR Primer Pairs | | | | | | | |
| --- | --- | --- | --- | --- | --- | --- | --- |
| Primer Name | Sequence | Len | Tm | GC. | Start | End | Pool |
| SLEV_1_LEFT | GGTGAGCGGAGAGGAAACAGAT | 22 | 62.05 | 54.55 | 13 | 35 | 1 |
| SLEV_1_RIGHT | TCCCTCCTCTCTTCTTGCTTGG | 22 | 61.42 | 54.55 | 414 | 392 | 1 |
| SLEV_2_LEFT | AGCCATCCTGACATTCTTCCGA | 22 | 61.48 | 50 | 241 | 263 | 2 |
| SLEV_2_RIGHT | CACCAACAGTCAATGTCCTCGG | 22 | 61.44 | 54.55 | 667 | 645 | 2 |
| SLEV_3_LEFT | CTAGTGCCAACGGAGCAAACAC | 22 | 62.25 | 54.55 | 537 | 559 | 1 |
| SLEV_3_RIGHT | ACCACTCTCTGTGTGTTGTTGC | 22 | 61.26 | 50 | 922 | 900 | 1 |
| SLEV_4_LEFT | GGACACCGTGAAAACCACCAAA | 22 | 61.78 | 50 | 796 | 818 | 2 |
| SLEV_4_RIGHT | CGTGTCCAAGGTTGCTTCGTAA | 22 | 61.37 | 50 | 1163 | 1141 | 2 |
| SLEV_5_LEFT | TACTTGAAGGGGGAAGCTGTGT | 22 | 61.3 | 50 | 1032 | 1054 | 1 |
| SLEV_5_RIGHT | CGTAGAGTCCGTTGAACCATGC | 22 | 61.55 | 54.55 | 1412 | 1390 | 1 |
| SLEV_6_LEFT | GCCTGTTTGGAAAAGGGAGCAT | 22 | 61.67 | 50 | 1278 | 1300 | 2 |
| SLEV_6_RIGHT | GCTCGTCCATGGAAGGTTCAAG | 22 | 61.51 | 54.55 | 1643 | 1621 | 2 |
| SLEV_7_LEFT | GGAACAGTTACCATTGATTGTGAAGC | 26 | 61.06 | 42.31 | 1511 | 1537 | 1 |
| SLEV_7_RIGHT | CAGTTCCACAATCACTGTCCCG | 22 | 61.44 | 54.55 | 1943 | 1921 | 1 |
| SLEV_8_LEFT | ATGCAGAGCTAAGCTTGACAAGG | 23 | 61.18 | 47.83 | 1822 | 1845 | 2 |
| SLEV_8_RIGHT | AAGCCTTACCAATGCTGCTTCC | 22 | 61.47 | 50 | 2184 | 2162 | 2 |
| SLEV_9_LEFT | GGGAGCGAACAACAAGGTCATG | 22 | 62.02 | 54.55 | 2056 | 2078 | 1 |
| SLEV_9_RIGHT | AGCCAGTAGAGTCAGCGAGATG | 22 | 61.59 | 54.55 | 2423 | 2401 | 1 |
| SLEV_10_LEFT | GTCCACCAAGTTTTCGGAGGAG | 22 | 61.13 | 54.55 | 2285 | 2307 | 2 |
| SLEV_10_RIGHT | CGTTGATCGGATGCCACAGATG | 22 | 61.93 | 54.55 | 2645 | 2623 | 2 |
| SLEV_11_LEFT | GGAGGAGGCATCTTCGTGTACA | 22 | 61.79 | 54.55 | 2510 | 2532 | 1 |
| SLEV_11_RIGHT | TCCATGCTCTGTTTGCTGTTGG | 22 | 61.65 | 50 | 2916 | 2894 | 1 |
| SLEV_12_LEFT | GCTGGAGGATGAATTGGACTACG | 23 | 61.05 | 52.17 | 2782 | 2805 | 2 |
| SLEV_12_RIGHT | CACCGGGATGATCATTTCGCTT | 22 | 61.32 | 50 | 3200 | 3178 | 2 |
| SLEV_13_LEFT | AGAGCAAAAAGAATGAGACATGGCA | 25 | 61.5 | 40 | 3072 | 3097 | 1 |
| SLEV_13_RIGHT | GGCCGGATTTCCATTCCATACC | 22 | 61.4 | 54.55 | 3475 | 3453 | 1 |
| SLEV_14_LEFT | AACATTGTGGAAACAGGGGAGC | 22 | 61.34 | 50 | 3330 | 3352 | 2 |
| SLEV_14_RIGHT | CCTCCAGTGTTCATTTCCGCAA | 22 | 61.39 | 50 | 3736 | 3714 | 2 |
| SLEV_15_LEFT | AAGCTGACCCTGACCTCACTAG | 22 | 61.15 | 54.55 | 3611 | 3633 | 1 |
| SLEV_15_RIGHT | GCCATCCTCATTCCTGGAGTCA | 22 | 61.82 | 54.55 | 4015 | 3993 | 1 |
| SLEV_16_LEFT | TGAAGCTTGAGGTCCTTCCGAT | 22 | 61.42 | 50 | 3882 | 3904 | 2 |
| SLEV_16_RIGHT | GCTATTGCAAAAGGGACCACCA | 22 | 61.41 | 50 | 4309 | 4287 | 2 |
| SLEV_17_LEFT | AAGTGAGGTCTTGACCGGAGTC | 22 | 61.66 | 54.55 | 4149 | 4171 | 1 |
| SLEV_17_RIGHT | AATGGAATCGTGCACTCAAGCC | 22 | 61.77 | 50 | 4539 | 4517 | 1 |
| SLEV_18_LEFT | CCCAGGCTTGATGTTGACCTTG | 22 | 61.72 | 54.55 | 4418 | 4440 | 2 |
| SLEV_18_RIGHT | CGGACATCTCCTGCATACGGAT | 22 | 61.98 | 54.55 | 4813 | 4791 | 2 |
| SLEV_19_LEFT | GGAGAAGGGAGACTAGATCCGT | 22 | 60.16 | 54.55 | 4711 | 4733 | 1 |
| SLEV_19_RIGHT | CTTTCCCCTTGGATGATGCCAC | 22 | 61.53 | 54.55 | 5104 | 5082 | 1 |
| SLEV_20_LEFT | CCGTGACGCTTGATTTCCCAAA | 22 | 61.96 | 50 | 4968 | 4990 | 2 |
| SLEV_20_RIGHT | GCCCTGATGTTCATTCCTCACG | 22 | 61.57 | 54.55 | 5357 | 5335 | 2 |
| SLEV_21_LEFT | ACACCAGCCGTGAAGAATGAAC | 22 | 61.39 | 50 | 5263 | 5285 | 1 |
| SLEV_21_RIGHT | CCTGGGCCTCAACATCCAGTAT | 22 | 61.82 | 54.55 | 5613 | 5591 | 1 |
| SLEV_22_LEFT | GCATTGCTGCTCGTGGGTATAT | 22 | 61.13 | 50 | 5475 | 5497 | 2 |
| SLEV_22_RIGHT | TGACTGGTTTCACACACTTGCG | 22 | 61.82 | 50 | 5895 | 5873 | 2 |
| SLEV_23_LEFT | GGAAAAGCTTTGACACAGAATACCCT | 26 | 61.69 | 42.31 | 5757 | 5783 | 1 |
| SLEV_23_RIGHT | ATCGAAACTCCCCATCCATGGT | 22 | 61.5 | 50 | 6168 | 6146 | 1 |
| SLEV_24_LEFT | CATGATCTGGCCAACTGGACTG | 22 | 61.26 | 54.55 | 6041 | 6063 | 2 |
| SLEV_24_RIGHT | ATGACTTGAGCGCTTGGTAGTC | 22 | 60.68 | 50 | 6423 | 6401 | 2 |
| SLEV_25_LEFT | GTGACTACCAAGCGCTCAAGTC | 22 | 61.49 | 54.55 | 6335 | 6357 | 1 |
| SLEV_25_RIGHT | CATCACAAATGCTCCCAGTCCC | 22 | 61.53 | 54.55 | 6710 | 6688 | 1 |
| SLEV_26_LEFT | GCTGGGAGCATTTGTGATGACT | 22 | 61.2 | 50 | 6627 | 6649 | 2 |
| SLEV_26_RIGHT | GGATGCGATGGCTGTTAGTGAG | 22 | 61.37 | 54.55 | 7060 | 7038 | 2 |
| SLEV_27_LEFT | ACTGGCTGTCTTCTTGATATGCA | 23 | 60.05 | 43.48 | 6844 | 6867 | 1 |
| SLEV_27_RIGHT | TTGGCAGGGTCATCTGATTCCA | 22 | 61.7 | 50 | 7233 | 7211 | 1 |
| SLEV_28_LEFT | TAACAGCCATTGCATCCCAAGC | 22 | 61.79 | 50 | 7110 | 7132 | 2 |
| SLEV_28_RIGHT | CTGCTGACCCTAAGACCCCAAA | 22 | 61.95 | 54.55 | 7518 | 7496 | 2 |
| SLEV_29_LEFT | GCGACCCCAATGACAGAGAAGAA | 23 | 62.74 | 52.17 | 7397 | 7420 | 1 |
| SLEV_29_RIGHT | GGGCTCTGTCTACTTCCACGAT | 22 | 61.53 | 54.55 | 7785 | 7763 | 1 |
| SLEV_30_LEFT | GAAAGCGTGGAGGAGGAAAAGG | 22 | 61.45 | 54.55 | 7662 | 7684 | 2 |
| SLEV_30_RIGHT | GGAACACGTCCACTCCACTTTT | 22 | 61 | 50 | 8070 | 8048 | 2 |
| SLEV_31_LEFT | CGCAACCCTGAAGCATGTTCAA | 22 | 62.22 | 50 | 7942 | 7964 | 1 |
| SLEV_31_RIGHT | GCCCCACTAACCCAGTACATCT | 22 | 61.49 | 54.55 | 8344 | 8322 | 1 |
| SLEV_32_LEFT | GTGTCCATACACGCCCAAAATCA | 23 | 61.67 | 47.83 | 8221 | 8244 | 2 |
| SLEV_32_RIGHT | TCACCATCGAGCTAGCTGATCC | 22 | 61.66 | 54.55 | 8640 | 8618 | 2 |
| SLEV_33_LEFT | AGTTGGGGAAAGGATACGGAGA | 22 | 60.56 | 50 | 8503 | 8525 | 1 |
| SLEV_33_RIGHT | TGGCTGTTCACCTTTGCTTTGA | 22 | 61.21 | 45.45 | 8893 | 8871 | 1 |
| SLEV_34_LEFT | ACCACTAGGAGTCGCCCAAATC | 22 | 62.06 | 54.55 | 8773 | 8795 | 2 |
| SLEV_34_RIGHT | CAAACTCCAAGAACCGAGCTCC | 22 | 61.44 | 54.55 | 9135 | 9113 | 2 |
| SLEV_35_LEFT | GATGGGAAAGCGCGAGAAGAAG | 22 | 61.87 | 54.55 | 8970 | 8992 | 1 |
| SLEV_35_RIGHT | ACCTTGTGGCGATAGGTCAGAT | 22 | 61.22 | 50 | 9339 | 9317 | 1 |
| SLEV_36_LEFT | TCCAGGAGGAAAGATGTACGCA | 22 | 61.15 | 50 | 9250 | 9272 | 2 |
| SLEV_36_RIGHT | TCAGGTCCATTCTTCCTCAGCC | 22 | 61.75 | 54.55 | 9643 | 9621 | 2 |
| SLEV_37_LEFT | CAACCTGGCCGTTCAACTGATA | 22 | 60.87 | 50 | 9517 | 9539 | 1 |
| SLEV_37_RIGHT | AGCTCATCCTGGCTCCTACATG | 22 | 61.61 | 54.55 | 9886 | 9864 | 1 |
| SLEV_38_LEFT | AGGACATTCAGGAGTGGAAACCT | 23 | 61.28 | 47.83 | 9750 | 9773 | 2 |
| SLEV_38_RIGHT | CTTCAATCCACACACGGTTCCA | 22 | 61.07 | 50 | 10143 | 10121 | 2 |
| SLEV_39_LEFT | GTTCAGCTGTCCCAGTCAACTG | 22 | 61.38 | 54.55 | 10023 | 10045 | 1 |
| SLEV_39_RIGHT | GCACTCCTCCTACCACATGAGT | 22 | 61.48 | 54.55 | 10386 | 10364 | 1 |
| SLEV_40_LEFT | CCACCTGGGCTGAGAACATCTA | 22 | 61.48 | 54.55 | 10254 | 10276 | 2 |
| SLEV_40_RIGHT | ACAGACAGCACCTTTAGCATGC | 22 | 61.45 | 50 | 10635 | 10613 | 2 |
| SLEV_41_LEFT | GTCAGGTAAACGGTGCTGTCTG | 22 | 61.43 | 54.55 | 10458 | 10480 | 1 |
| SLEV_41_RIGHT | CTGGTGTTGAAAAAGCAGGGGA | 22 | 61.28 | 50 | 10884 | 10862 | 1 |

# Table S3: BEAST Model Testing using generalized stepping-stone sampling

|  | TN93 + G4 | |
| --- | --- | --- |
| Molecular Clock | Strict Molecular Clock | Relaxed Molecular Clock Lognormal |
| Constant Population | -17052.83 | -17011.81 |
| Exponential Population | -17049.65 | -17010.90 |
| Skyline | -17029.09 | **-16702.81** |
| Skygrid | -17050.94 | -21298.41 |
| Skyride | -17062.33 | -17003.13 |
